# Frizzled 7 modulates goblet and Paneth cell fate, and maintains homeostasis in mouse intestine

**DOI:** 10.1242/dev.200932

**Published:** 2023-02-14

**Authors:** Nai-Xin Gu, Yu-Ru Guo, Sey-En Lin, Yen-Hsin Wang, I.-Hsuan Lin, Yi-Fan Chen, Yun Yen

**Affiliations:** ^1^ The PhD Program for Translational Medicine, College of Medical Science and Technology, Taipei Medical University and Academia Sinica, Taipei 11529, Taiwan; ^2^ Graduate Institute of Cancer Biology and Drug Discovery, College of Medical Science and Technology, Taipei Medical University, Taipei 11031, Taiwan; ^3^ Department of Anatomic Pathology, New Taipei Municipal Tucheng Hospital, Chang Gung Memorial Hospital and Chang Gung University, New Taipei City 236017, Taiwan; ^4^ Graduate Institute of Translational Medicine, College of Medical Science and Technology, Taipei Medical University, Taipei 11031, Taiwan; ^5^ TMU Research Center of Cancer Translational Medicine, Taipei Medical University, Taipei 11031, Taiwan; ^6^ Master Program in Clinical Genomics and Proteomics, School of Pharmacy, Taipei Medical University, Taipei 11031, Taiwan; ^7^ International Ph.D. Program for Translational Science, College of Medical Science and Technology, Taipei Medical University, Taipei 11031 , Taiwan; ^8^ Ph.D. Program for Cancer Molecular Biology and Drug Discovery, College of Medical Science and Technology, Taipei Medical University, Taipei 11031, Taiwan; ^9^ Cancer Center, Taipei Municipal WanFang Hospital, Taipei 116081 , Taiwan

**Keywords:** Frizzled 7, Goblet/Paneth cells, Intestinal epithelium, Inflammation, Tumorigenesis

## Abstract

Intestinal homeostasis depends on interactions between the intestinal epithelium, the immune system and the microbiota. Because of these complicated connections, there are many problems that need to be solved. Current research has indicated that genes targeted by Wnt signaling are responsible for controlling intestinal stem cell fate and for modulating intestinal homeostasis. Our data show that loss of frizzled 7 (Fzd7), an important element in Wnt signaling, interrupts the differentiation of mouse intestinal stem cells into absorptive progenitors instead of secretory progenitors (precursors of goblet and Paneth cells). The alteration in canonical Wnt and Notch signaling pathways interrupts epithelial homeostasis, resulting in a decrease in physical protection in the intestine. Several phenotypes in our Fzd7-deleted model were similar to the features of enterocolitis, such as shortened intestines, decreased numbers of goblet cells and Paneth cells, and severe inflammation. Additionally, loss of Fzd7 exacerbated the defects in a chemical-induced colitis model and could initiate tumorigenesis. These findings may provide important information for the discovery of efficient therapeutic methods to treat enterocolitis and related cancers in the intestines.

## Teaser

**Summary:** Frizzled 7 deletion interrupts the differentiation of stem cells and changes the cell distribution of intestinal epithelium, which drives tissue damage in intestines.

## INTRODUCTION

The intestinal epithelium includes several distinct cell populations, such as intestinal stem cells, Paneth cells, goblet cells, enterocytes, enteroendocrine cells, microfold cells, cup cells and tuft cells (van der Flier and Clevers, 2009). These cells coordinate with each other and perform various functions, including digestion, absorption, physical or cell-mediated immunity, and regular renewal. Homeostatic maintenance in the intestines is modulated by genetic factors, extrinsic factors, the immune system, mucosal barriers and the microbiota distribution (Maloy and Powrie, 2011). Loss of integrity and homeostasis in the intestinal epithelium are crucial factors in enterocolitis and other subsequent diseases, as well as cancers (Maloy and Powrie, 2011; Scoville et al., 2008; Okumura and Takeda, 2018).

The maintenance of intestinal homeostasis also depends on communication between epithelial cells and non-epithelial cells (Koch, 2017). One group of non-epithelial cells are immune cells, which are located in or recruited close to the intestines, and play a crucial role in maintaining intestinal homeostasis (Liang et al., 2006). The crosstalk between epithelial and immune cells occurs through cytokine generation and secretion or cell-cell contact (Pull et al., 2005; Heller et al., 2005, 2008). Immune cells also modulate physical barriers, e.g. by affecting mucus generation, and chemical barriers in the intestines. Barriers efficiently segregate the microbiota and host cells to maintain a symbiotic relationship in the intestines. Some reports have also indicated that inflammatory responses are caused by a dysfunction in the mucus barrier, which is produced by epithelial cells (Jostins et al., 2012; Maloy and Powrie, 2011). Therefore, defects in either intestinal epithelial cells or immune cells may destroy homeostasis in the intestines and lead to various disorders and diseases (Okumura and Takeda, 2017).

The intestinal epithelial layer provides the major line of defense against damage by and invasion of pathogens. Many intrinsic and extrinsic factors can destroy the homeostasis of the intestinal epithelium, resulting in various intestinal diseases. Enterocolitis is a disorder with inflammatory responses causing enteritis of the intestinal tract, including the small intestine, colon and rectum. Severe inflammation contributes to the pathogenesis of necrotizing enterocolitis, inflammatory bowel disease and cancers. To date, there have been no efficient therapeutic methods developed for humans with these diseases. It is still necessary to explore the key factors involved in the pathogenic mechanisms of diseases and discover new efficient therapeutic methods.

*Fzd7* encodes a protein with seven transmembrane domains that functions as a receptor of Wnt proteins and facilitates the activation of downstream targets in the Wnt signaling pathway (Finch et al., 1997). Fernandez et al. reported higher expression of Fzd7 in undifferentiated cells than in differentiated cells, and, accordingly, Wnt signals are important for maintaining the undifferentiated state of stem cells (Fernandez et al., 2014). Wnt signals are transduced through frizzled receptors to initiate canonical or noncanonical pathways (Katoh and Katoh, 2007b; Niehrs, 2012). Wnt signals modulate self-renewal, metabolism, survival, proliferation and epithelial-to-mesenchymal transition in target cells. Active Wnt signaling is essential for maintaining epithelial homeostasis through crosstalk with the BMP, FGF, Hedgehog (HH), Notch and transforming growth factor β (TGFβ) signaling pathways (Miyoshi, 2017; Kuhnert et al., 2004). These signaling pathways coordinate with each other to control epithelial homeostasis.

Previous reports indicate that Fzd7 participates in stem cell regeneration and tumor development. Fzd7 has been reported to be highly expressed in intestinal stem cells (leucine-rich repeat-containing G protein-coupled receptor 5+, Lgr5+) (Flanagan et al., 2015). Inhibition of Fzd7 expression in Lgr5+ cells could impair stem cell functions in organoids and a mouse model (Flanagan et al., 2015; Nile et al., 2018). Fzd7 is also involved in the pathogenesis of various types of cancer, such as hepatocellular carcinoma, breast cancer, squamous cell carcinoma, lung cancer, cervical cancer, ovarian cancer, glioma and melanoma (Zeng et al., 2018). Fzd7 overexpression is highly associated with advanced tumor stages in gastric and colorectal cancers, although the studies reporting these finding were performed using human tumor tissues and *in vitro* culture systems (Geng et al., 2016; Ueno et al., 2009; Li et al., 2018).

Members of the Frizzled class receptor family transduce signals from Wnt proteins into cells, which contributes to the control of tissue development and regeneration. In this study, we observed several features present during the pathogenesis of enterocolitis in a conventional Fzd7 knockout (Fzd7 KO) model, but these features were not observed in a stem cell (Lgr5+)-specific Fzd7 knockout model in a previous report (Flanagan et al., 2015). Most interestingly, Fzd7 deletion changes the distribution of intestinal epithelial cells. We also report that Fzd7 not only plays a crucial role in epithelial renewal, but may also have an important effect on intestinal immunity. The combined effects of regenerative defects and immune dysregulation (chronic inflammation) could elevate the incidence of intestinal diseases.

## RESULTS

### Shortened length and delayed regeneration of Fzd7-deleted intestines

**Fig. 1.**
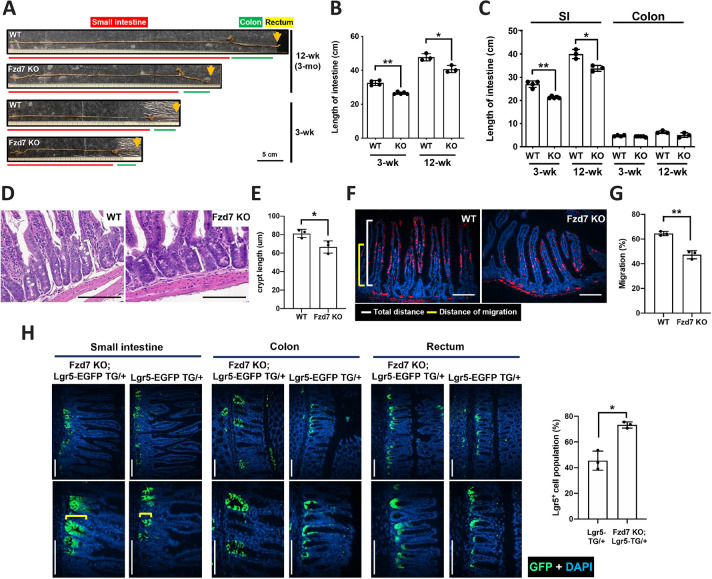
**Phenotypic characterization of the intestines of Fzd7 KO mice.** (A) Gross view of the intestines at 3 and 12 weeks (3 months). (B) Length of the intestines in mice at 3 weeks and 12 weeks (3 months). (C) Length of the small intestines (SI) and colon in mice at 3 weeks and 12 weeks (3 months). (D) Crypt length in the small intestines of mice at 3 months. (E) Quantified data of crypt length in the small intestines of mice. Data are mean±s.d. Measurements were taken from at least nine crypts for each sample. *n*=3 mice per group. (F) EdU staining for cell proliferation in mice at 3 months. (G) Quantification of migrated EdU^+^ cells in the small intestines of mice. Migration distance was defined as the distance from villus bottom to the foremost EdU-positive cell. Data are mean±s.d. Measurements were taken from an average of 10 villi for each sample. *n*=3 mice per group. (H) Tracing of Lgr5-expressed cells (GFP^+^ cells) in the small intestine, colon and rectum of Fzd7-KO mice with the Lgr5-EGFP-Cre transgene at 3 months. The distribution of Lgr5^+^ cells in the small intestine of mice at 3 months calculated in relation to the length of crypt. Measurements were taken from an average of seven crypts for each sample (*n*=3 mice per group). In B and C, *n*=4 mice per group for wild-type and *n*=5 mice per group for Fzd7 KO at 3 weeks, and *n*=3 mice per group at 12 weeks. Data are mean±s.d. *P<0.05; **P<0.01 (two-tailed unpaired *t*-tests). Scale bars: 100 μm in D,F,H.

The intestinal epithelial layer needs to perform daily self-renewal to maintain functionality. The epithelial and mesenchymal layers are modulated by various signaling pathways to maintain homeostasis in the intestines. Wnt signaling pathways play important roles in intestinal homeostasis, and Fzd7 is one of the key factors. We postulate that Fzd7 and related molecular mechanisms play important roles in the intestinal homeostasis; therefore, Fzd7 deletion may interrupt the renewal and functions of the intestines. An Fzd7 KO mouse model was designed and generated as shown in Fig. S1A-C. The expression levels of *Fzd7* were obviously low in the whole intestines (small intestine, colon and rectum) and peripheral blood cells of Fzd7 KO mice (Fig. S1D,E). Interestingly, the length of the whole intestines was shorter in Fzd7 KO mice than in wild-type mice, particularly the length of the small intestine (Fig. 1A-C). However, there were no effects on body weight or metabolic indices (food and water intake, urine, and stool production) in Fzd7 KO mice (Fig. S1F,G). The intestinal epithelium is continuously renewed every day and various functional cells (villi) are differentiated from stem cells (bottom of the crypt). Crypt length was obviously shortened in Fzd7 KO mice (Fig. 1D,E), and the rate of cell proliferation was also decreased because of the relatively low migration rate of EdU-positive cells (the distributed length of EdU-positive cells versus the length of a whole villus) (Fig. 1F,G). Lgr5 is a marker for tracing intestinal stem cells. We generated Fzd7 KO mice expressing an *EGFP* transgene driven by *Lgr5* promoter (Fig. S2A-C), and, surprisingly, more Lgr5-positive cells were shown to accumulate in the crypts of Fzd7 KO mice than in those of wild-type mice (Fig. 1H). We also checked another intestinal stem cell marker, Olfm4, the expression levels of which were not significantly altered in the Fzd7 KO mice (Fig. S2D,E). The data revealed that loss of Fzd7 along the crypt-villus axis slowed cell migration and interrupted crypt extension but had no noticeable effects on villus extension. We posited that Fzd7 deletion may disturb the cell differentiation of the intestinal epithelium, resulting in feedback modulating the proliferation of intestinal stem cells.

### Fzd7 deletion interrupts the homeostasis of the intestinal epithelium

**Fig. 2.**
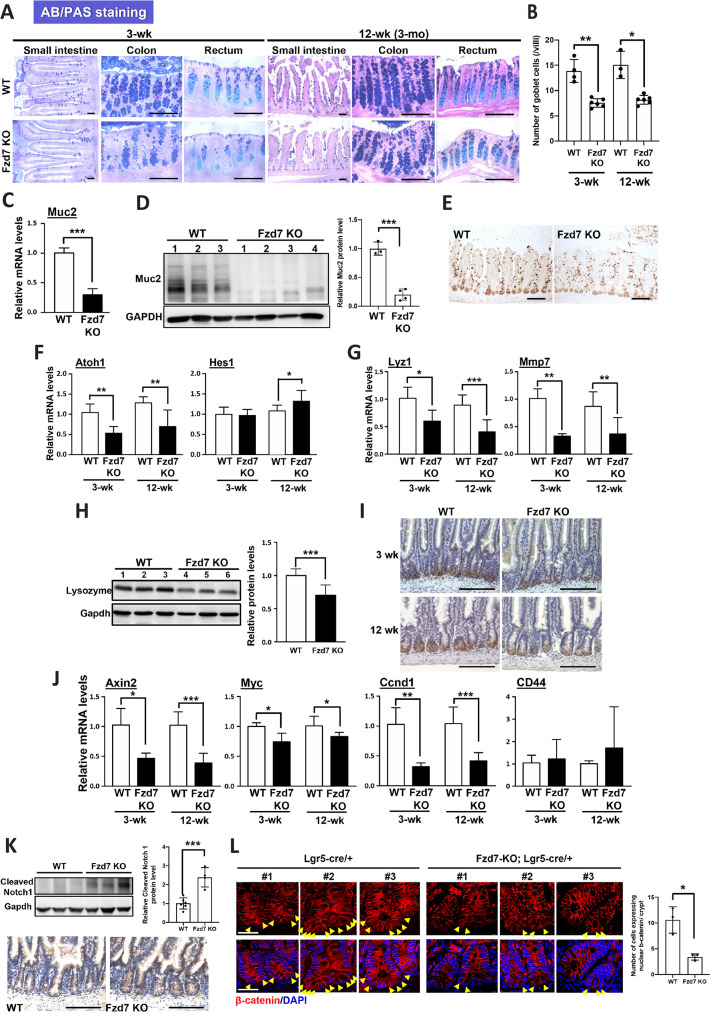
**A decrease in goblet cells and Paneth cells in the intestines of Fzd7 KO mice.** (A) AB/PAS combined staining was performed on the small intestine, colon and rectum of 3- and 12-week-old (3-month-old) mice. (B) Quantitative data of goblet cell numbers in the intestinal sections. Measurements were taken from an average of 40 villi for each sample. *n*=4 and *n*=3 mice for wild-type groups at 3 weeks and 12 weeks, respectively. *n*=6 mice for both Fzd7 KO groups. (C) The expression levels of *Muc2* (goblet cell marker) were detected in the small intestine of mice at 3 weeks by RT-qPCR. (D) The protein level of Muc2 was detected in the small intestine of mice at 3 months. Protein levels were calculated relative to Gapdh as a control. Wild-type mice, *n*=3; Fzd7 KO mice, *n*=4. (E) Immunohistochemistry staining of Muc2 was performed on the small intestine of 3-month-old mice. (F) The expression levels of *Atoh1* and *Hes1* (for goblet cell maturation and involved in the Notch signaling, respectively) were detected in the small intestine of mice by RT-qPCR. Wild-type mice, *n*=6; Fzd7 KO mice, *n*=6. (G) The expression levels of *Lyz1* and *Mmp7* (a Paneth cell marker) were detected in the small intestine of mice by RT-qPCR. Wild-type mice, *n*=6; Fzd7 KO mice, *n*=6. (H) The protein levels of lysozyme were detected in the small intestine of mice at 3 months. Protein levels were calculated relative to Gapdh as a control. Wild-type mice, *n*=5; Fzd7 KO mice, *n*=10. (I) IHC staining of lysozyme was performed on the small intestine of mice. (J) The expression changes of genes involved in the Wnt canonical signaling pathway were detected in the small intestine of mice. Wild-type mice, *n*=6; Fzd7 KO mice, *n*=6. (K) The expression levels of cleaved Notch 1 were analyzed using western blot analysis and IHC analysis. Wild-type mice, *n*=6; Fzd7 KO mice, *n*=4. (L) The IF staining of β-catenin in intestines were performed and the number of β-catenin-expressing nuclei per crypt was quantified. Measurements were taken from at least 16 crypts for each sample. Yellow arrowheads indicate cells with β-catenin-expressing nuclei. Wild-type mice, *n*=3; Fzd7 KO mice, *n*=3. PAS, periodic acid-Schiff (neutral mucus staining); AB, Alcian Blue (acid mucus staining). Data are mean±s.d. *P<0.05; **P<0.01; ***P<0.001 (two-tailed unpaired *t*-tests). Scale bars: 100 μm in A,E,I,K; 50 μm in L.

Wnt signaling is responsible for controlling the proliferation and differentiation of stem cells. Fzd7 deletion can delay the differentiation process of intestinal stem cells, so whether the population and functions of differentiated cells in the intestinal epithelium are also affected in Fzd7 KO mice remains unclear. The number of goblet cells was dramatically decreased in the whole intestines, including the small intestine, colon and rectum of Fzd7 KO mice (Fig. 2A,B; Fig. S3A). Mucin 2, a marker of goblet cells, had low expression in the Fzd7 KO small intestine (Fig. 2C-E) but not in the Fzd7 KO colon (Fig. S3B). The levels of *Atoh1* and *Hes1*, which are involved in goblet cell maturation, were also significantly decreased in the Fzd7 KO small intestine (Fig. 2F; Fig. S3C). *Lyz1* and *Mmp7*, which are markers for Paneth cells, showed robust decreases in mRNA and protein expression in the Fzd7 KO small intestine (Fig. 2G-I); however, similar changes did not occur in Fzd7 KO colon (Fig. S3D). Additionally, the decreased numbers of goblet and Paneth cells were accompanied by increases in the numbers of enterocytes and enteroendocrine cells in Fzd7 KO mice (Fig. S3E-G). Therefore, our data demonstrate that Fzd7 deficiency not only disrupts the differentiation process of intestinal stem cells but also alters the differentiation rate of different cell types and cell populations in the intestinal epithelium.

### Fzd7 deficiency interrupts signaling pathways involved in cell proliferation, differentiation and migration

The functions of intestinal stem cells, including self-renewal, proliferation and differentiation, are tightly controlled by numerous regulatory pathways. Notably, events downstream of the canonical Wnt signaling pathway were disrupted in the small intestine of Fzd7 KO mice (Fig. 2J); a similar result was observed in the Fzd7 KO colon, except for increased expression of *Cd44* (Fig. S4A). Our data indicated that β-catenin could not be activated and translocated into the nucleus of intestinal epithelial cells in Fzd7 KO mice (Fig. S4B). Notch signaling can promote the differentiation of intestinal stem cells into absorptive progenitors (precursors of enterocytes) instead of secretory progenitors (precursors of goblet and Paneth cells); therefore, interruption of Notch signals could contribute to the higher percentage of enterocytes and lower percentages of goblet and Paneth cells in Fzd7 KO intestines. Atoh1 was dominantly downregulated through Wnt signaling pathway and indeed the low expression of β-catenin was observed in the nucleus of Fzd7 KO intestines; meanwhile upregulated Notch signaling increased Hes1 expression (Fig. 2K,L). The BMP signaling pathway also participates in regulating the differentiation of intestinal stem cells. However, in Fzd7 KO mice, the expression of key factors involved in the BMP signaling pathway was not obviously different from that in wild-type mice (Fig. S4C,D). Therefore, our data indicate that dysregulation of canonical Wnt and Notch signals along the crypt-villus axis disturbs the renewal and differentiation of the intestinal epithelium in Fzd7-deleted mice.

### Severe inflammation observed in the Fzd7 KO mouse model

**Fig. 3.**
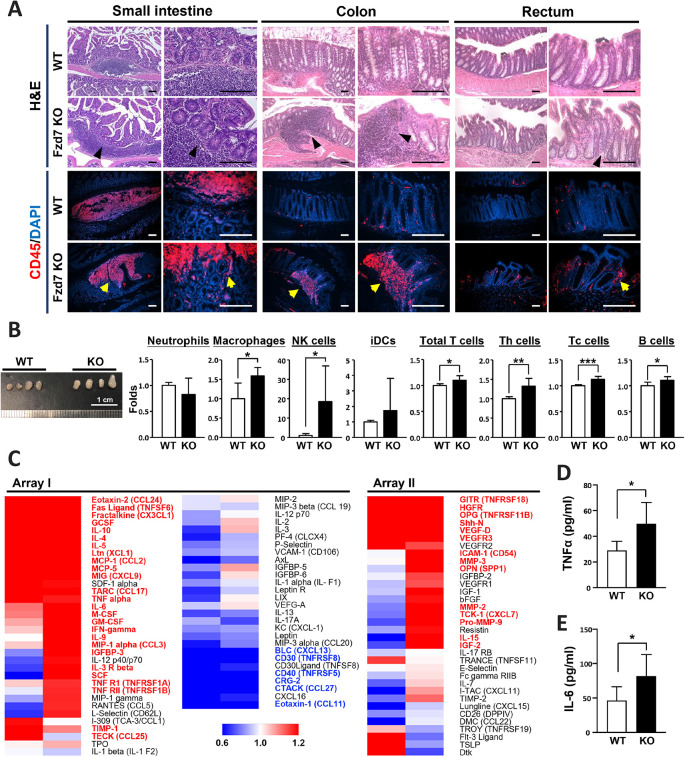
**Inflammatory responses were observed in the intestines of Fzd7 KO mice.** (A) The locations of Payer's patches and inflammatory response stained using Hematoxylin and Eosin in mice. The CD45 immunofluorescent (IF) staining indicates the accumulation and infiltration of leukocytes. DAPI is a counterstain for IF staining. All tissues were obtained from 3-month-old mice. Black and yellow arrowheads indicate tissues with immune cell infiltration. (B) Analysis of various immune cell populations in the inguinal lymph nodes of mice. Wild-type mice, *n*=7; Fzd7 KO mice, *n*=5. *P<0.05; **P<0.01; ***P<0.001 (two-tailed unpaired *t*-tests). (C) Heat map shows the increase or decrease of cytokine levels in Fzd7 KO mice. RayBio C-Series Mouse Cytokine Antibody Array Kit was used to evaluate the expression levels of various cytokines. Array I is the probed membrane for RayBio C-Series Mouse Cytokine Antibody Array C3 (monitoring 62 mouse proteins). Array II is the probed membrane for RayBio C-Series Mouse Cytokine Antibody Array C4 (monitoring 34 mouse proteins). (D) The TNFα levels in intestinal cell extracts were detected using ELISA analysis. Wild-type mice, *n*=5; Fzd7 KO mice, *n*=5. (E) The IL6 levels in intestinal cell extracts were detected using ELISA analysis. Wild-type mice, *n*=6; Fzd7 KO mice, *n*=7. Data are mean±s.d. *P<0.05 (two-tailed unpaired *t*-test). Scale bars: 100 μm.

Fzd7 deletion interferes with intestinal regeneration and differentiation, contributing to the pathogenesis of enterocolitis. We also proposed that Fzd7 deletion may disturb the functions of immune cells and the distribution of the intestinal microbiota; therefore, Fzd7 deletion not only causes defects in the regenerative capacity and immune responses but also interrupts the crosstalk between these processes. Immune cells in Peyer's patches are responsible for immune responses in the mucosa of the intestines. Increased immune cells were observed in the whole intestines of Fzd7 KO mice (Fig. 3A). The populations of immune cells in the inguinal lymph nodes in Fzd7 KO mice were examined (Fig. S5A), and the levels of populations involved in cell-mediated immune responses, such as natural killer (NK) cells, helper T cells and cytotoxic T cells, were elevated (Fig. 3B). The proportions of B cells and macrophages in the lymph nodes were slightly but significantly increased in Fzd7 KO mice (Fig. 3B). A cytokine assay revealed that crucial pro-inflammatory cytokines, including TNFα, IL6, IFNγ, MCP1 and MIP1α, were highly expressed in the Fzd7 KO mice. Additionally, the levels of several chemokines, such as eotaxin 2 (CCL24), fractalkine (CX3CL1), Ltn (XCL1), MCP1 (CCL2), MCP5 (CCL12), MIG (CXCL9), TRC (CCL17) and MIP1α, were elevated in the circulation of Fzd7 KO mice; these chemokines are responsible for recruiting T cells, NK cells and macrophages to inflamed tissues (Fig. 3C; Fig. S5B). We also verified the expression of key pro-inflammatory cytokines, TNFα and IL6, and they were upregulated in Fzd7 KO mice (Fig. 3D,E). Additionally, the NF-κB signaling, one crucial pathway that modulates inflammation, was upregulated in Fzd7 KO mice (Fig. S5C). These cytokines and chemokines could be secreted from damaged tissues and surrounding immune cells. As we predicted, the microbiota distribution was changed in the Fzd7 KO mice (Fig. S6). Accordingly, we conclude that cytokine expression is highly correlated with the immune cell population present in the Fzd7-deleted condition.

### Fzd7-deleted mice had similar phenotypes to the dextran sulfate sodium-treated mice

**Fig. 4.**
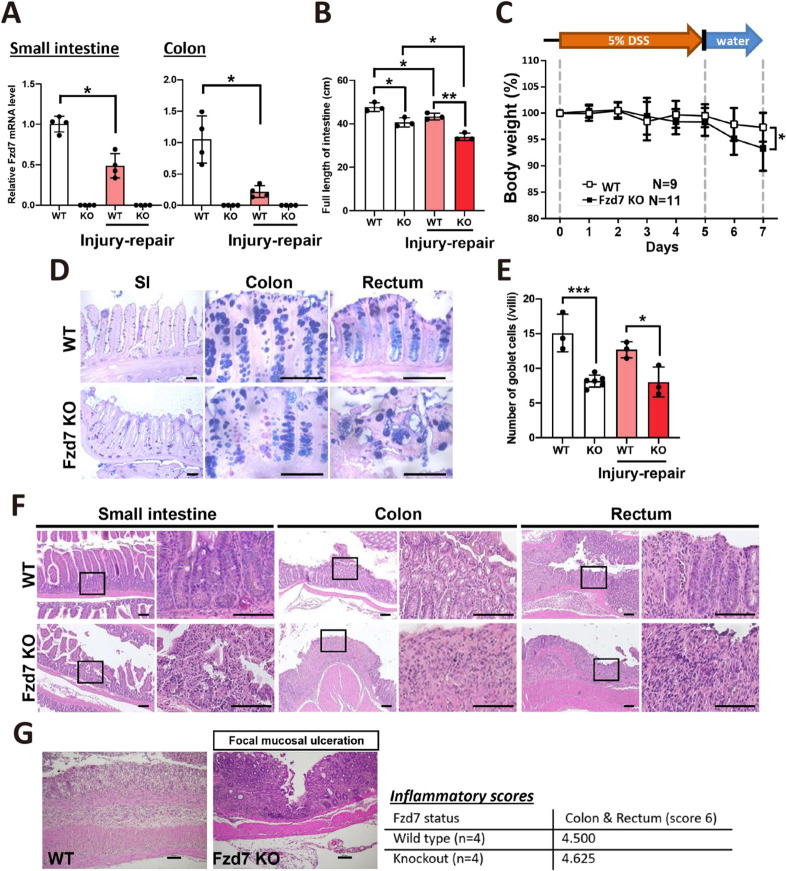
**Shortened intestines and inflammation in acute injury-repair models with Fzd7 deletion.** (A) The mRNA expression levels of *Fzd7* in the small intestine and colon in mice after acute injury-repair treatment. *n*=4 mice per group. (B) Quantification of length of the intestines in mice after acute injury-repair treatment. *n*=3 mice per group. (C) The body weight of mice during acute injury and repair process. Two-tailed unpaired *t*-tests were performed at each time point. Wild-type mice, *n*=9; Fzd7 KO mice, *n*=11. (D) PAS-AB staining of the intestines (small intestine, colon and rectum) in 3-month-old mice after acute injury-repair treatment. (E) Quantification of goblet cell numbers in intestines after acute injury-repair treatment. *n*=6 for non-treated Fzd7 KO group; *n*=3 for all other groups. Measurements were taken from an average of 40 villi for each sample. (F) Histopathological analysis of the intestines (small intestine, colon and rectum) in 3-month-old mice after acute injury-repair treatment. (G) Diffuse mucosal ulceration and inflammatory infiltration in the mucosa and submucosa were induced by acute DSS injury in 3-month-old wild-type mice. Focal mucosal ulceration and transmural inflammation with inflammatory infiltration in the perirectal fat tissue were induced by acute DSS injury in Fzd7 KO mice. Data are mean±s.d. *P<0.05; **P<0.01; ***P<0.001 (two-tailed unpaired *t*-tests). Scale bars: 100 μm.

Previous reports have indicated that treatment with relatively low molecular weight dextran sulfate sodium (DSS) (5 kDa) results in relatively mild colitis but severe inflammatory responses in the colon, rectum and small intestine (Lykov et al., 2018; Umiker et al., 2019; Scheibe et al., 2019; Rehal et al., 2018). After DSS treatment (acute injury-repair model), as described in Fig. S7A, wild-type mice had decreased *Fzd7* expression in the small intestine and colon (Fig. 4A). Interestingly, both Fzd7 deletion and acute DSS treatment caused intestinal shortening and inflammatory responses (Fig. 4B; Fig. S7B,C), but the body weight of Fzd7 KO mice was lower than that of wild-type mice (Fig. 4C). Although the number of goblet cells was not significantly changed after DSS treatment, a disorganized and distorted pattern of goblet cells was observed in Fzd7 KO mice (Fig. 4D,E); additionally, more vacuoles were observed in the rectum of Fzd7 KO mice than that of wild-type mice (Fig. S7D). After DSS treatment, wild-type intestines regenerated and recovered completely, but Fzd7 deletion disturbed and delayed the regenerative process, and induced inflammatory responses (Fig. 4F). The colonic mucosa showed disruption of the epithelium with mixed inflammatory cell infiltration in the lamina propria and ulceration in the mucosa or submucosa (Fig. 4G); however, the inflammatory scores (calculated as described by Erben et al., 2014) of Fzd7 KO and wild-type mice were not obviously different in the acute phase (Fig. 4G). Fzd7 deletion and DSS treatment probably had different effects on the canonical Wnt signaling pathway (Fig. S7E,F), but they caused synergistic effects that exacerbated intestinal phenotypes and enhanced inflammatory responses.

### Severe inflammatory infiltration and mucosal regeneration changes in Fzd7 KO mice with chronic injury

**Fig. 5.**
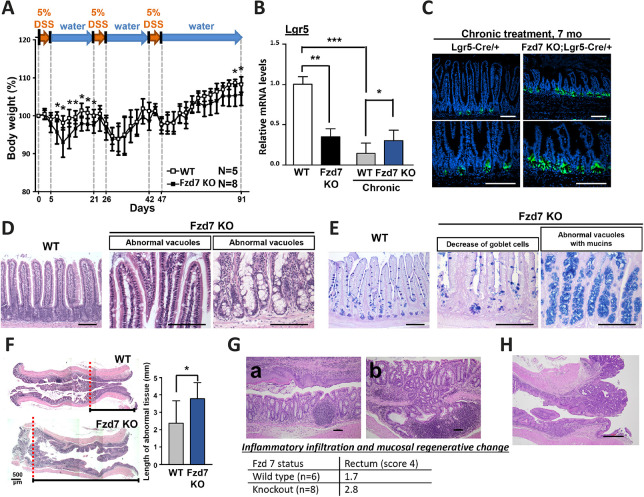
**More-severe phenotypes were observed in Fzd7 KO mice with chronic inflammation.** (A) The body weight of mice was monitored after chronic DSS treatment. Two-tailed unpaired *t*-tests were performed at each time point. Wild-type mice, *n*=5; Fzd7 KO mice, *n*=8. (B) The mRNA expression levels of *Lgr5* were detected in the small intestine of mice with or without chronic DSS treatment. Tissues were harvested at 7 months. *n*=6 mice per group. (C) The EGFP signals indicated the Lgr5-positive cells in the small intestine after chronic DSS treatment. Scale bars: 100 μm. (D) The abnormal vacuoles were observed in the small intestine of Fzd7 KO mice after chronic DSS treatment using Hematoxylin and Eosin staining. Scale bars: 100 μm. (E) The abnormal features in the small intestine of Fzd7 KO mice after chronic DSS treatment using AB/PAS staining. Scale bars: 100 μm. (F) Obvious expansion of squamous metaplasia in the rectum of mice after chronic DSS treatment. Wild-type mice, *n*=5; Fzd7 KO mice, *n*=8. (G) Rectal adenocarcinoma developed in the Fzd7 KO mouse with chronic DSS injury. Scale bar: 500 μm. (H) Fzd7 KO mice with chronic DSS treatment had several phenotypes, such as extensive and diffuse infiltration, and mucosal regenerative change. Scale bars: 500 μm. (a) and (b) are two individual mice. Data are mean±s.d. *P<0.05; **P<0.01; ***P<0.001 (two-tailed unpaired *t*-tests).

Severe damage to tissues induces continuous regeneration reactions and chronic inflammatory responses, which both trigger and accelerate pathogenesis in the intestines. Fzd7 modulates intestinal epithelial regeneration and immune homeostasis, and deletion of this gene increases the probability of inducing tumor growth. To induce chronic inflammation, Fzd7 KO mice were given long-term DSS treatment, as presented in Fig. S8A. The body weight of the Fzd7 KO mice was not rapidly restored during the recovery period after the first DSS treatment and was noticeably lower than that of wild-type mice at the end of treatment (Fig. 5A). After chronic DSS injury, the expression levels of Lgr5 remained low in Fzd7 KO mice, whereas they dramatically decreased in the wild-type mice (Fig. 5B); accordingly, a decrease in the frequency of Lgr5-positive cells was observed in the wild-type mice after chronic DSS treatment (Fig. 5C; Fig. S8B). Abnormal histopathology was observed in the small intestine of Fzd7 KO mice with chronic DSS injury, including a decrease in the number of goblet cells and many abnormal vacuoles with mucins (Fig. 5D,E). However, chronic DSS treatment had no additional effects on the distribution or differentiation of the intestinal epithelium (Fig. S8C-F). The genes involved in canonical Wnt signaling and goblet cell maturation were also not changed between mice with or without chronic DSS injury (Fig. S8G,H). Interestingly, after chronic DSS injury, the rectal areas showed re-epithelialization with squamous metaplasia, especially in Fzd7 KO mice (Fig. 5F). In addition, several phenotypes were also observed in Fzd7 KO mice given chronic DSS treatment, such as (1) diffuse mucosal and submucosal inflammatory infiltration and marked squamous metaplasia, and (2) marked mucosal and submucosal inflammatory infiltration with submucosal herniation and goblet cell depletion (Fig. 5G); moreover, Fzd7 deletion elevated the inflammatory score in the rectum (Fig. 5G). In our study, Fzd7 KO mice with chronic DSS injury for only 90 days had the potential to develop rectal adenocarcinoma (1/8=12.5%) (Fig. 5H). Consequently, the combination of an intrinsic defect (Fzd7 deletion) and extrinsic factor (chronic DSS injury) seems to exhaust intestinal damage and contribute to tumorigenesis.

### Fzd7 deletion disturbs the distribution of microbiota and further induces liver tumorigenesis

**Fig. 6.**
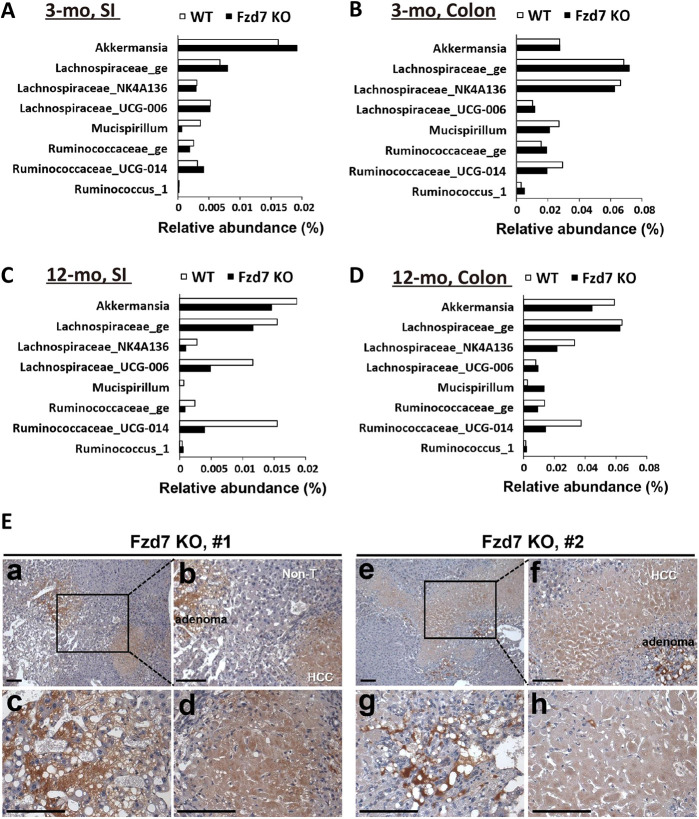
**The microbiota distribution and further influence on the liver of the Fzd7 KO mice.** Distinct small intestine (SI) and colon microbiota profiles at genus level of mice. (A) Microbiome composition of the intestines in mice at 3 months. (B) Microbiome composition of the colon in mice at 3 months. Wild-type mice, *n*=8; Fzd7 KO mice, *n*=9. (C) Microbiome composition of the intestines in mice at 12 months. (D) Microbiome composition of the colon in mice at 12 months. All data are presented as mean relative abundance. Wild-type mice, *n*=2; Fzd7 KO mice, *n*=2. (E) IHC staining with antibodies against β-catenin was performed for the observation of hepatocellular adenoma in mice. b is the area indicated in a; f is the area indicated in e. c and g are adenoma parts; d and h are hepatocarcinoma (HCC) parts. Scale bars: 100 μm.

A decrease in the number of goblet cells leads to a lower amount of mucus for protecting the intestinal epithelium, which induces immune cell infiltration and changes microbiota distribution. As we know, microbial dysbiosis and released metabolites could induce liver inflammation and fibrosis contributing to liver tumorigenesis (Gupta et al., 2019). Additionally, prominent changes in gut microbiotas lead to outgrowth of bacteria, dysbiosis and intestinal permeability, resulting in the liver pathogenesis and further hepatocellular carcinoma (HCC); for example, the alteration of *Akkermansia*, Lachnospiraceae, *Mucispirillum* and Ruminococcaceae in guts (Liu et al., 2019; Jia et al., 2021; Rattan et al., 2020; Wang et al., 2020; Xia et al., 2018; Zheng et al., 2020; Loy et al., 2017; Qian et al., 2020). Based on our microbiota analysis, we observed the changes of these microbiotas in Fzd7 KO mice (Fig. 6A-D). The decreased amounts of *Akkermansia*, Lachnospiraceae and Ruminococcaceae could reduce the intestine immunosuppression and increase inflammation in 12-month-old Fzd7 KO mice (Fig. 6C,D). *Mucispirillum* could reduce the ability to scavenge oxygen and reactive oxygen species, and enhance inflammatory responses (Loy et al., 2017). An increase of *Mucispirillum* was observed in the colon of 12-month-old Fzd7 KO mice (Fig. 6D). As recent studies have shown that disturbances of intestinal microbiota and the metabolites are involved in the progression of chronic liver diseases (Llorente and Schnabl, 2015; Hartmann et al., 2019; Jones and Neish, 2021), these alterations of intestinal microbiotas could re-disturb the intestinal functions and enhance several features in liver, such as spotty necrosis (7/9=77.78%), portal inflammation (8/9=88.89%), piecemeal necrosis (6/9=66.67%), hepatocyte dysplasia (4/9=44.44%), focal coagulative necrosis (1/9=11.11%), fatty metamorphosis (2/9=22.22%) and further tumor growth (2/9=22.22%) in 12-month-old  Fzd7 KO mice (Fig. 6E; Fig. S9A-C). However, there was no significantly different histopathological pattern in livers between Fzd7 KO and wild-type mice at 5, 7 and 9 months of age (Fig. S9D). Therefore, the imbalance of intestinal microbiota may not only destroy the intestinal integrity but also induce the liver pathogenesis in Fzd7 KO mice.

## DISCUSSION

**Fig. 7.**
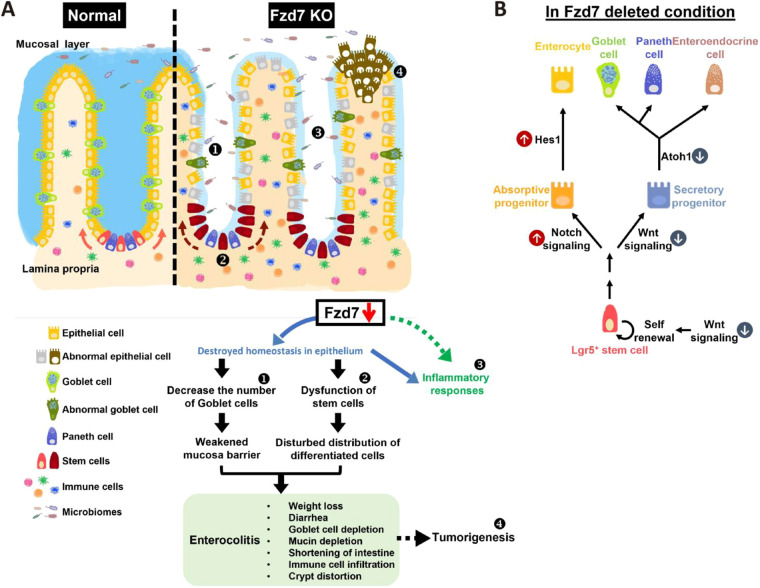
**Fzd7 contributes to maintain the homeostasis of intestinal epithelium and its microenvironment.** (A) Fzd7 deficiency causes several effects as follows: (1) interfering with the development and maintenance of goblet cells; (2) blocking the regenerative capacity of intestinal stem cells; (3) modulating inflammatory reactions and destroying microbiota homeostasis; and (4) inducing the features of enterocolitis and initiating the process for tumor formation. (B) In the Fzd7-deleted condition, which is unbalanced between the Wnt and Notch signaling pathways, there is a decrease in secretory cells and an increase in absorptive cells.

The features of enterocolitis include shortened intestines, decreased numbers of goblet cells and Paneth cells, and severe inflammation, all of which contribute to tumorigenesis in the intestines. Our results indicate that Fzd7 is involved in the maintenance of homeostasis in the intestines, such as epithelial renewal and differentiation, and immune modulation. Fzd7 deletion interrupted the differentiation of stem cells and changed the distribution of cell types in the intestinal epithelium. Goblet cells are thought to secrete mucins to form the mucus layer to protect the intestinal epithelium; however, in the Fzd7-deleted condition, more enterocytes and enteroendocrine cells were differentiated than goblet cells, resulting in reduced mucus protection of the intestines. Additionally, opportunistic infections by intestinal microbiota might occur accompanied by elevated inflammatory responses. Increased macrophage numbers, as shown by our data, could be responsible for eliminating microbes that crossed the disrupted epithelial barrier into the villi. Fzd7 has functions in intestinal stem cells and other epithelial cells, as well as in immune cells; therefore, immune cells might be affected directly by Fzd7 deletion. All these phenotypes drive the process of enterocolitis, and related features were observed in Fzd7 KO mice. We also postulated that Fzd7 deletion indirectly initiates tumorigenesis in the intestines, and our results show that Fzd7 deficiency (intrinsic defect) combined with DSS treatment (extrinsic factor) can induce adenocarcinoma in mice. Conclusively, Fzd7 has important functions in maintaining the regular differentiation and homeostasis of the intestinal epithelium, and loss of Fzd7 function is a crucial issue in the pathogenesis of enterocolitis, as well as in the formation of tumors (Fig. 7A). We propose that Fzd7 deletion leads to an imbalance between Wnt and Notch signaling pathways that results in the alteration of the distribution of intestinal epithelium (Fig. 7B).

Previous reports indicated that Fzd7 deletion in Lgr5-positive cells reduced the regenerative capacity of the intestines (Flanagan et al., 2015). The Fzd7 genetically modified animal models used were two conditional knockout mice using AhCre (Cre recombinase driven by cytochrome P450 promoter; Ireland et al., 2004) and Lgr5^EGFP-IRES-CreERT2^, respectively and one conventional knockout mouse at 6-12 weeks. Flanagan and colleagues thoroughly investigated the effects of Fzd7 deletion on intestinal stem cells and indicated that Fzd7 plays an important role in robust Wnt-dependent processes in intestinal stem cells (Lgr5-positive cells). The models they used were analyzed at ∼12 weeks of age and did not show any phenotypes related to enterocolitis. In human patients, the onset and progression of disease are induced by systemic problems, such as genetic defects or environmental stress. Enterocolitis is induced by an imbalance between proinflammatory and anti-inflammatory processes, disturbed epithelial homeostasis and a changed distribution of the microbiota. The Fzd7 conventional knockout model, which was generated in our lab, was analyzed from 3 weeks to 12 months of age to confirm that Fzd7 deficiency causes severe enterocolitis and other phenotypes with time. As our data show, both isolated intestinal epithelium and circulated immune cells have Fzd7 expression (Fig. S1E); therefore, Fzd7 deletion may have effects on whole intestinal epithelial cells other than stem cells.

In addition to canonical Wnt signals, several signaling pathways coordinate with each other to control intestinal epithelial homeostasis. The BMP signaling pathway, in opposition to Wnt signals, inhibits intestinal stem cell activation and promotes intestinal differentiation (Kosinski et al., 2007; Du et al., 2015). Several factors in the FGF family are target genes in canonical Wnt signaling in the gastrointestinal tract (Katoh and Katoh, 2006). Similar to Wnt signals, the Hedgehog signaling pathway plays important roles in intestinal crypt-villus axis formation and stem cell homeostasis (Madison et al., 2005). The TGFβ and Wnt signaling pathways have no common factors; however, some reports indicate that TGFβ signaling interferes with the control of Wnt signals in cells in the intestines, such as stem cells and goblet cells (McCauley et al., 2014; Cammareri et al., 2017). The Notch and Wnt signals coordinately control stem cell homeostasis and the balances of secretory and absorptive cell lineage differentiation (Du et al., 2015; Tian et al., 2015); these two pathways work together to maintain stem cell functions, proliferation and differentiation (Andreu et al., 2005; van Es et al., 2005; Zecchini et al., 2005). Wnt pathway impairment interrupts Paneth cell differentiation in individuals with Crohn's disease (Wehkamp et al., 2007; O'Brien et al., 2007; Schepers et al., 2012). Obviously, Fzd7 deletion reduced canonical Wnt and induced Notch signaling, but did not change other pathways. This deletion seemed to show no compensatory effects on signal transduction in the Fzd7 KO model. As our data showed, the level of *Cd44*, which is downstream of the Wnt pathway, was increased in the colon of Fzd7 KO mice (Fig. S4A). Colorectal cancer stem cells exhibit enhanced CD44, CD133 and Lgr5 expression (Zeilstra et al., 2008); therefore, loss of Fzd7 may initiate tumorigenesis in the colon.

Wnt signaling pathways are highly interconnected with several major inflammatory pathways. Wnt signals intersect with the NF-κB signaling pathway: a major inflammatory pathway that contributes to modulating cell proliferation, cell survival and differentiation (Ma and Hottiger, 2016). A previous report indicated that activation of Wnt signaling inhibits the NF-κB pathway, because β-catenin interacts with NF-κB to block the binding of p65 and p50 (subunits of NF-κB) to DNA (Deng et al., 2002). On the other hand, NF-κB can inhibit TCF/β-catenin-dependent transcription in intestinal epithelial cells; by contrast, a NF-κB and β-catenin interaction occurs during intestinal inflammation and drives epithelial dedifferentiation (Schwitalla et al., 2013; Cho et al., 2008). Notably, loss of Fzd7 reduced the expression of downstream Wnt signals, as well as β-catenin, and upregulated TNFα expression might activate NF-κB signaling, both of which contribute to exacerbating the epithelial defects and inflammatory responses. However, the intersection between the Wnt and NF-κB signaling pathways involved in modulating intestinal stem cell function and immune responses is complicated and needs to be verified in the future.

DSS-injured mice showed a destroyed epithelium, crypt distortion, goblet cell depletion, submucosal oedema and inflammatory cell infiltration in the intestines. Blocking the Notch pathway at an early stage could recover the loss of goblet cells and ameliorate DSS-induced colitis (Shinoda et al., 2010). In addition, neutralization of TNFα, a proinflammatory cytokine, could restore goblet cell function and alleviate disease progression (Dharmani et al., 2011). After DSS treatment, reduced expression of Fzd7 was detected in the intestines of wild-type mice (as shown in Fig. 4C), and mice with Fzd7 deletion had phenotypes similar to those presented in DSS-injured mice. Accordingly, the signaling or mechanism involving Fzd7 may be a crucial issue in inducing the features of DSS-induced damage in mice.

Microorganisms in the gut, i.e. the intestinal microbiota, are mostly considered beneficial for their hosts, but some of these microbes pose a potential threat to the host. The thick mucus layer covering the intestinal epithelium prevents the translocation of microbes. Loss of mucus generation weakens immune protection of the intestinal epithelium, which can then be invaded more easily by intestinal microbes. Alterations in the intestinal environment have very large effects on the diversity and number of microbes. Therefore, it will be necessary to analyze the microbiota distribution in the Fzd7 KO model in the future.

Inflammatory bowel diseases (IBDs), comprising Crohn's disease (CD) and ulcerative colitis (UC), are inflammatory diseases in the intestines, characterized by injury of the intestinal epithelium and chronic relapsing inflammation. Epithelial barrier dysfunction is pivotal in the disease progression of IBD (McGuckin et al., 2009). Individuals with CD exhibit defects in Paneth and goblet cells, but those with UC mostly exhibit goblet cell depletion and decreased MUC2 production (Adolph et al., 2013; Van Klinken et al., 1999; Gersemann et al., 2009). The defects in goblet and Paneth cell differentiation in both diseases may allow intestinal microbe invasion and subsequent elevations in inflammatory responses (Gersemann et al., 2011). Goblet and Paneth cells are modulated by Wnt and Notch pathways during differentiation (Gregorieff and Clevers, 2005; Katoh and Katoh, 2007a). The features of IBD could also be observed in the Fzd7 KO model. We postulate that the loss of Fzd7 has a synergistic effect that exacerbates the disease progression of both CD and UC. The dysbiosis and microbiota-related metabolites lead to a dysfunctional mucosal immunity and an increase in intestinal permeability (Quraishi et al., 2017). The gut microbiota regulated inflammatory responses and contributed to hepatocarcinogenesis (Schwabe and Greten, 2020).

Overall, loss of Fzd7 perturbs the differentiation of the goblet and Paneth cell lineages, and promotes commitment to the enterocyte lineage, ultimately contributing to the induction of inflammatory responses and the progression of diseases. Defects in Wnt signaling in Fzd7-deleted conditions alter Notch signaling and may activate the NF-κB pathway to interfere with intestinal epithelial homeostasis and enhance inflammatory responses. A genetic defect (intrinsic) and dietary choice (extrinsic) can have synergistic effects that promote disease progression; therefore, our study provides a clue towards the mechanism underlying the onset and progression of disorders in the intestines, and suggests new avenues to explore for the development of effective therapeutic methods.

## MATERIALS AND METHODS

### Mouse model

Mouse genomic *Fzd7* DNA was obtained by screening the BAC library (RP23-425A4/RP24-510I17). *Fzd7* exon 1, which is part of a Not I-Spe I genomic DNA fragment (29.8 kb), was inserted into the PL253 vector (Fig. S1A). The linear Fzd7 targeting fragment was transferred into embryonic stem (ES) cells (JM8A3, B6N agouti). Targeted ES clones were screened with neomycin and injected into blastocysts (B6J). A final mouse model with conventional Fzd7 deletion and a mouse model with *Fzd7* floxed alleles were obtained. In order to trace the population of stem cells, Fzd7 KO mice were mated with Lgr5-EGFP-IRES-CreERT2 mice (008875, The Jackson Laboratory) to generate the Fzd7 KO mice with Lgr5-GFP transgene. All animal protocols were approved by Institutional Animal Care and Use Committee (IACUC) of Taipei Medical University and National Defense Medical Center.

### RNA analysis

Total RNA was isolated from the intestines using TRIzol Reagent (Thermo Fisher Scientific). Real-time quantitative PCR was performed using TaqMan probes with the TaqMan Universal PCR Master Mix (Roche Life Science). Amplification was executed in triplicate for each RNA sample and primer set. Primers and probes are listed in Table S1.

### Protein analysis

Tissue samples were homogenized in lysis buffer [20 mM Tris (pH 7.4), 150 mM NaCl, 10 mM EDTA and 1% Triton X-100 with a complete protease inhibitor cocktail (Roche)] and then denatured in SDS sample buffer in boiling water. The total extracted proteins were separated on a 10% SDS polyacrylamide gel (Bio-Rad) and electro-transferred to a Hybond N+ membrane (GE Healthcare). Membranes were blocked with 5% (w/v) non-fat dry milk, incubated with primary antibodies against lysozyme (1:1000, Dako, A0099), NF-κB-p65 (1:1000, Cell Signaling Technology, 8242), IκBα (1:1000, 9242, Cell Signaling Technology), p-IκBα (1:1000, Cell Signaling Technology, 9246), cleaved-Notch1 (1:1000, Cell Signaling Technology, 4147) and glyceraldehyde-3-phosphate dehydrogenase (Gapdh, 1:5000, GeneTex, GTX100118), and finally detected using the Visualizer Kit (Millipore).

### Histopathology

The intestinal tissues were obtained by the following step. The front end and rear end, which account for one-ninth of the total length were removed. The imaging of the intestine was mainly from middle part to distal part, which are approximately equivalent to jejunum and ileum. Tissues were fixed in formalin buffer and embedded in paraffin wax. Tissue sections (3 µm) were subjected to Haematoxylin and Eosin, immunohistochemical (IHC), immunofluorescence (IF) and special staining following standard procedures. IHC staining was performed by soaking sections in antigen-retrieval buffer containing 10 mM sodium citrate (pH 6.0) and heating the sections in a microwave oven for 10 min twice. The sections were then incubated with primary antibodies against lysozyme (1:1000, Dako, A0099) and Smad4 (1:1000, Cell Signaling Technology), β-catenin (1:500, BD Biosciences, 610153), Notch1 (1:100, Abcam, ab52627) and macrophages (1:100, Abcam, ab125148) detected with Dako REAL EnVision/HRP secondary antibodies (1:50, Dako, K5007), and visualized with the Dako REAL EnVision Detection System (Dako, K5007). IF staining was carried out with primary antibodies, including antibodies targeting CD45 (1:500, BD Biosciences, 550539), Green fluorescent protein (GFP, 1:500, GeneTex, GTX113617), β-catenin (1:500, BD Biosciences, 610153) and Olfm4 (1:400, Cell Signaling Technology, 39141), and Alexa Fluor 488- or Alexa Fluor 568-labelled secondary antibodies (1:500, Thermo Fisher Scientific, A-10680 and A-11004). For visualization of nuclei, tissues were counterstained with 4'-6-diamidino-2-phenylindole (DAPI, Thermo Fisher Scientific, D1306) and mounted in fluorescence mounting medium (Thermo Fisher Scientific). Fluorescent images were obtained using Stellaris 8 confocal microscope (Leica). Periodic acid-Schiff (PAS)-Alcian Blue (MUTO, No. 40931, No. 4085-1) staining was performed on intestinal tissues to observe the location of goblet cells.

### EdU staining

5-Ethynyl-2′-deoxyuridine (EdU) is a thymidine analogue. Two and 48 h before sacrifice, mice were given EdU (300 μg, Thermo Fisher Scientific) by intraperitoneal injection to monitor intestinal epithelial cell proliferation and migration by specific staining. Tissue sections were fixed with 4% paraformaldehyde and incubated with Proteinase K (800 ng/μl) for permeabilization, followed by addition of a Click-iT Plus TUNEL Reaction cocktail on tissues. For visualization of nuclei, slides were counterstained with DAPI (4′-6-diamidino-2-phenylindole, Thermo Fisher Scientific, D1306) and mounted in fluorescence mounting medium (Thermo Fisher Scientific).

### Flow cytometry

Three-month-old wild-type mice and Fzd7 knockout mice were sacrificed for experimental evaluation. The cells in the inguinal lymph nodes were collected and resuspended in RPMI-1640 medium. After washing twice with staining buffer (BD), the cells from the inguinal lymph nodes (1×10^6^) were stained with different antibodies (anti-CD11b, anti-CD11c, anti-CD3, anti-CD4, anti-CD45, anti-CD8a, anti-CD19, anti-F4/80, anti-Gr-1, anti-MHCII and anti-NK1.1) listed in Fig. S5A. All cells were passed through 100 μm pore nylon mesh and analyzed on a BD FACSVerse flow cytometer.

### Cytokine assay

The RayBio C-Series Mouse Cytokine Antibody Array Kit (RayBiotech) was used to evaluate the expression levels of various cytokines. The antibody array was carefully removed from the plastic packaging, and each membrane was placed into a well of the incubation tray and incubated with 2 ml of blocking buffer for 30 min at room temperature. The blocking buffer was then removed from each well, and the wells were incubated with serum or plasma at 4°C overnight. Following aspiration of the samples from each well, 1 ml of detection antibody was added for 2 h at room temperature followed by addition of 2 ml of HRP-streptavidin concentrate for 2 h at room temperature. Three washes were performed between each incubation step. Detection buffer was added to each membrane and incubated for 2 min at room temperature. Each array membrane was observed and analyzed by a chemiluminescence imaging system. Calculated data values higher than 1.2 or lower than 0.6 indicated a significant difference between two groups.

### Enzyme-linked immunosorbent assay (ELISA)

Tissue samples were homogenized in lysis buffer [20 mM Tris (pH 7.4), 150 mM NaCl, 10 mM EDTA and 1% Triton X-100 with a complete protease inhibitor cocktail (Roche)]. TNFα and IL6 levels in intestinal cell extracts were measured by the Mouse TNF-α Quantikine HS ELISA Kit (MHSTA50, R&D Systems) and Mouse IL-6 Quantikine ELISA Kit (M6000B, R&D Systems), according to the manufacturer's protocols. The results were analyzed using a Synergy H4 Reader (BioTek Instruments).

### Targeted amplicon library preparation and sequencing

Targeted amplification of the bacterial *16S rRNA* gene and library construction were performed according to Illumina's recommended protocols (https://support.illumina.com/downloads/16s_metagenomic_sequencing_library_preparation.html). Briefly, the universal primers 341F (5′-CCTACGGGNGGCWGCAG-3′) and 805R (5′-GACTACHVGGGTATCTAATCC-3′) containing Illumina overhang adaptor sequences in the forward (5′-TCGTCGGCAGCGTCAGATGTGTATAAGAGACAG-3′) and reverse (5′-GTCTCGTGGGCTCGGAGATGTGTATAAGAGACAG-3′) primers were used to amplify the V3-V4 region of the bacterial *16S rRNA* gene using a limited cycle PCR. Next, the Illumina sequencing adaptors and dual-index barcodes were attached to the amplicon targets using a Nextera XT Index kit (Illumina). A QSep100 analyzer (BiOptic) was then used to check the quantity and quality of the sequencing libraries. Finally, the libraries were normalized and pooled in an equimolar ratio and sequenced on a MiSeq (Illumina) using v3 chemistry to generate paired-end reads of 300 bases in length.

### *16S rRNA* gene sequencing and statistical analysis

Universal primer sequences and low-quality reads were trimmed with cutadapt (v1.15) (Martin, 2011). The trimmed reads were processed and analyzed with the DADA2/phyloseq workflow in the R environment. Briefly, filtering, trimming, dereplication and denoise of the forward and reversed reads were performed with the DADA2 package (v1.6) (Callahan et al., 2016). Overlapping paired-end reads were merged and chimeras were detected and removed to obtain a clean set of inferred amplicon sequence variants (ASVs). Taxonomic assignment of the ASVs was performed using the SILVA reference database (v132) (Quast et al., 2013) with a minimum bootstrap confidence of 80. Multiple sequence alignment of the ASVs was performed with DECIPHER package (v2.6.0) (Wright, 2015) and a phylogenetic tree was constructed using RAxML (v8.2.11) (Stamatakis, 2014). The frequency table, taxonomy and phylogenetic tree information were used to create a phyloseq object. Bacterial community analysis was performed using the phyloseq package (v1.22.3) (McMurdie and Holmes, 2013). The bacterial compositions of samples were visualized using bar plots. Alpha diversity indices were calculated using the ‘estimate_richness’ function of the phyloseq package. UniFrac distances were calculated using the GUniFrac package (v1.1) to assess community dissimilarity between groups (Chen et al., 2012). Principal coordinate analysis (PCoA) ordination on UniFrac distances was performed using the phyloseq package.

### Dextran sulfate sodium treatment

Dextran sulfate sodium (DSS) salt is a highly water-soluble compound and 5.0% DSS in drinking water was prepared to cause intestinal injury. There were two groups in our study. In our injury-repair model, 5% DSS in the drinking water was administered for the first 5 days, followed by administration of regular drinking water for 2 days. A chronic model was established with 5% DSS in the drinking water administered for the first 5 days, followed by administration of regular drinking water for 16 days. The DSS-water cycle was repeated three times, and regular drinking water was provided for 1 month after the last cycle. Mouse body weight was measured daily.

### Statistical analysis

The data are presented as mean±s.d. Comparisons between the two groups were calculated using a two-tailed Student's *t*-test. Statistical differences between two groups were analyzed, and the differences were considered significant when *P*<0.05.

## Supplementary Material

10.1242/develop.200932_sup1 Supplementary information
