# Supplementary material for: Frizzled 7 modulates goblet and Paneth cell fate, and maintains homeostasis in mouse intestine
Source: Development. 2023 Feb 14;150(4):dev200932. doi: 10.1242/dev.200932 (PMC10112897; doi:10.1242/dev.200932)
Supplement: Supplementary information [file develop-150-200932-s1.pdf]

Supplemental Figure S1

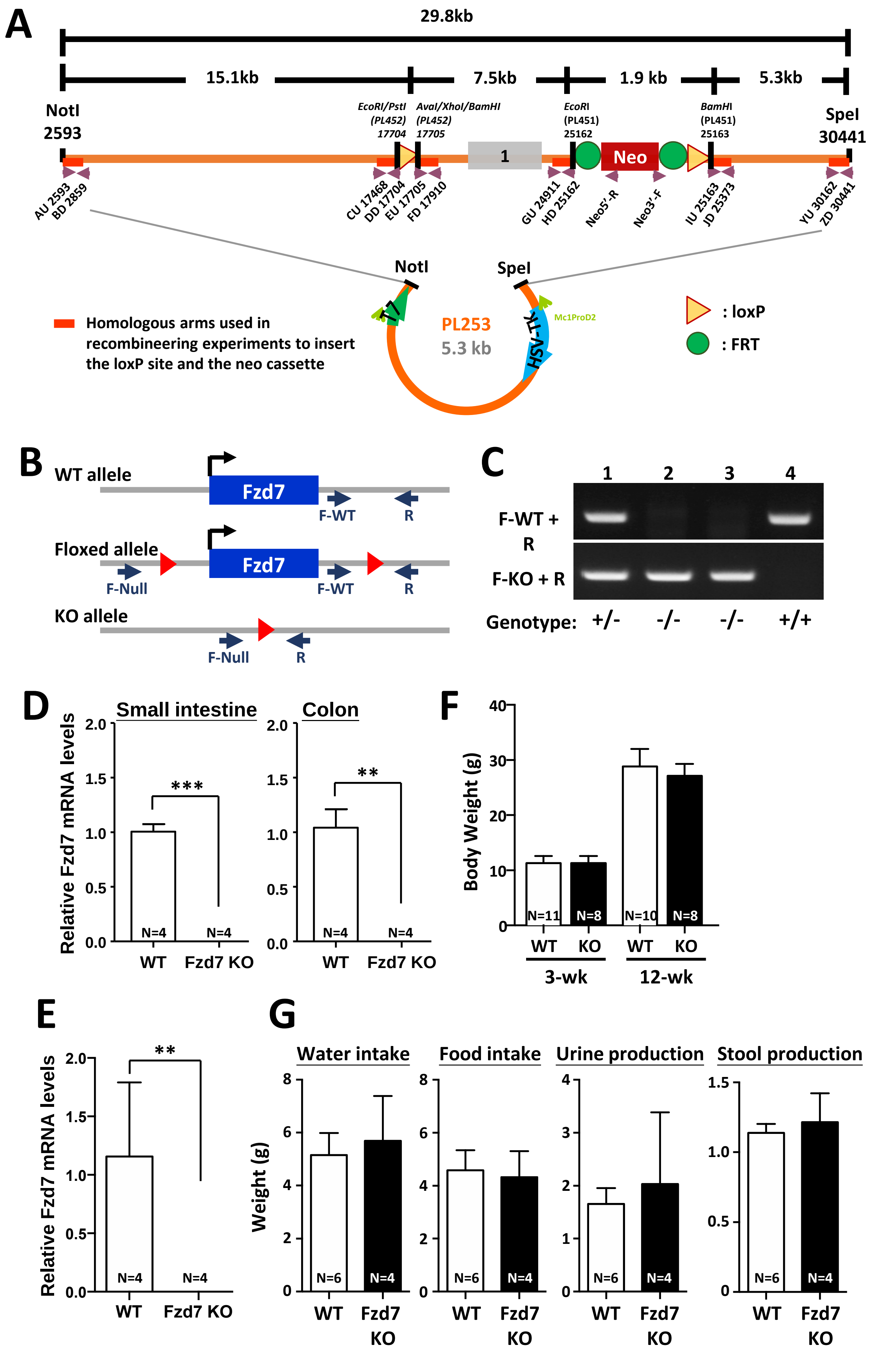

Development • Supplementary information

Fig. S1. Generation of Fzd7 conventional knockout mouse model. (A) Construction of Fzd7 in PL253 plasmid. The design and funder generation were operated and purchased by Transgenic mouse models core facility (A4), National Core Facility for Biopharmaceuticals, MOST. Neo, neomycin. **(B)** Primers designed for distinct wild-type (WT) allele, Floxed allele and knockout (KO) allele. F, forward primer; R, reversed primer. **(C)** The fragment amplified by primer F-WT and primer R indicates this mouse has WT allele. The fragment amplified by primer F-Null and primer R indicates this mouse has KO allele. **(D)** The expression levels of Fzd7 in intestines were detected by RT-qPCR analysis. Mouse age was 3 months old. **(E)** The expression levels of Fzd7 in peripheral blood mononuclear cells were detected by RT-qPCR analysis. Mouse age was 3 months old. **(F)** Body weight of Fzd7 KO and WT mice were monitored at 3 months old. **(G)** The metabolic indexes were measured using metabolic cages in Taiwan Mouse Clinic. Mouse age was 3 months old. The results are presented as the mean ± SD. ***p*<0.01; ****p*<0.001.

Development • Supplementary information

Supplemental Figure S2

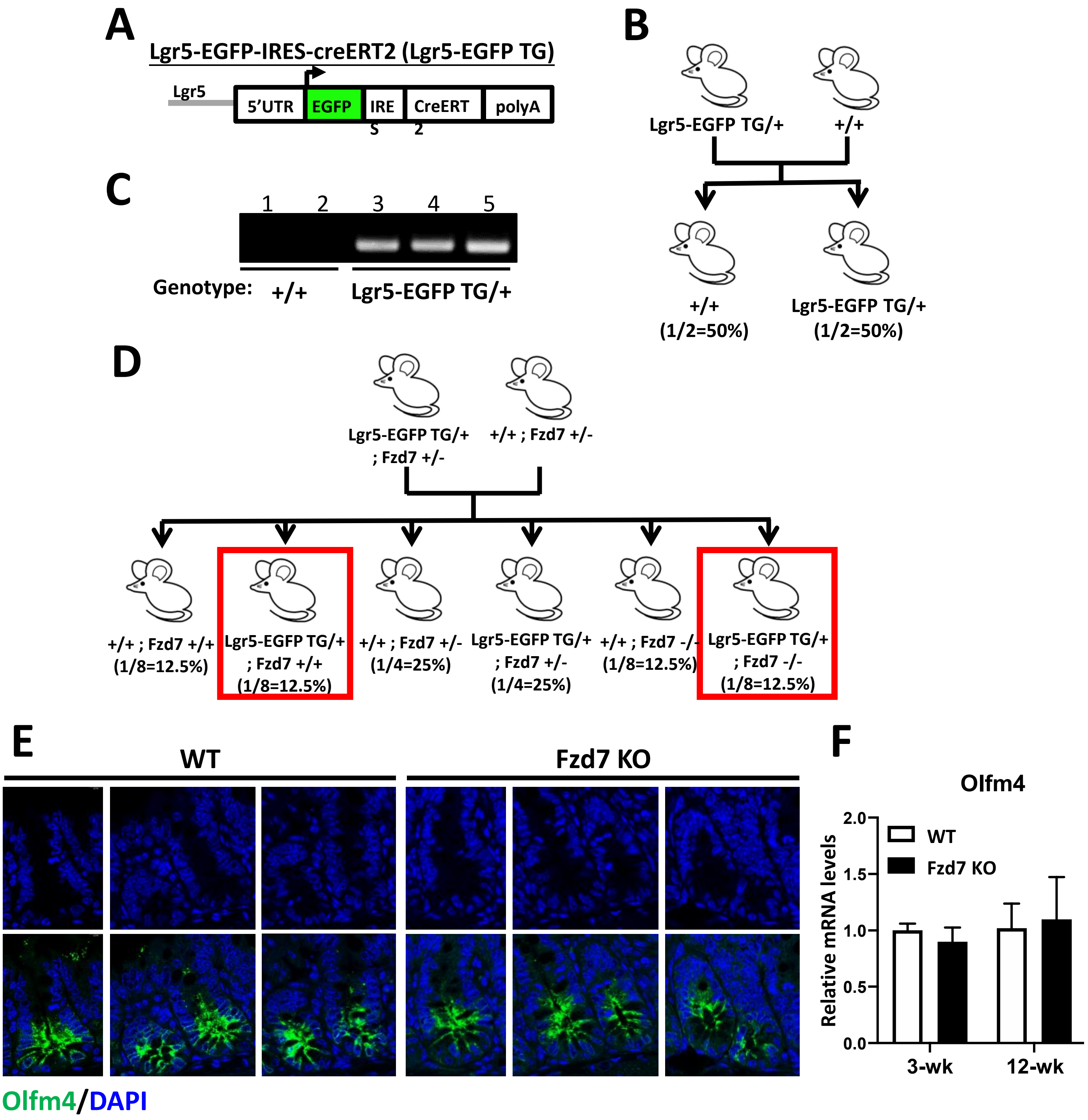

Fig. S2. Construction and breeding strategies Lgr5-EGFP-IRES-creERT2 knock-in mouse with Fzd7 deletion. (A) Construct of Lgr5-EGFP-IRES-creERT2 knock-in mouse obtained from JAX Lab. The figure was modified from the construction described in original reference (Barker N. et al., 2007) **(B)** Breeding strategy for Lgr5-EGFP-IRES-creERT2 knock-in mouse model. **(C)** Genotyping of Lgr5-EGFP-IRES-creERT2 knock-in mouse was detected by PCR analysis. **(D)** Breeding strategy for Lgr5-EGFP-IRES-creERT2 knock-in mouse with Fzd7 homologous knockout mouse. **(E)** The IF staining of Olfm4, an intestinal stem cell marker. Green, Olfm4; Blue, DAPI. **(F)** The Olfm4 mRNA expression levels in the Fzd7 KO and WT mice. There were 4-10 mice in each group. The results are presented as the mean ± SD.

Reference

Barker N, van Es JH, Kuipers J, Kujala P, van den Born M, Cozijnsen M, Haegebarth A, Korving J, Begthel H, Peters PJ, Clevers H. 2007. Identification of stem cells in small intestine and colon by marker gene Lgr5. Nature 449(7165):1003-7.

Supplemental Figure S3

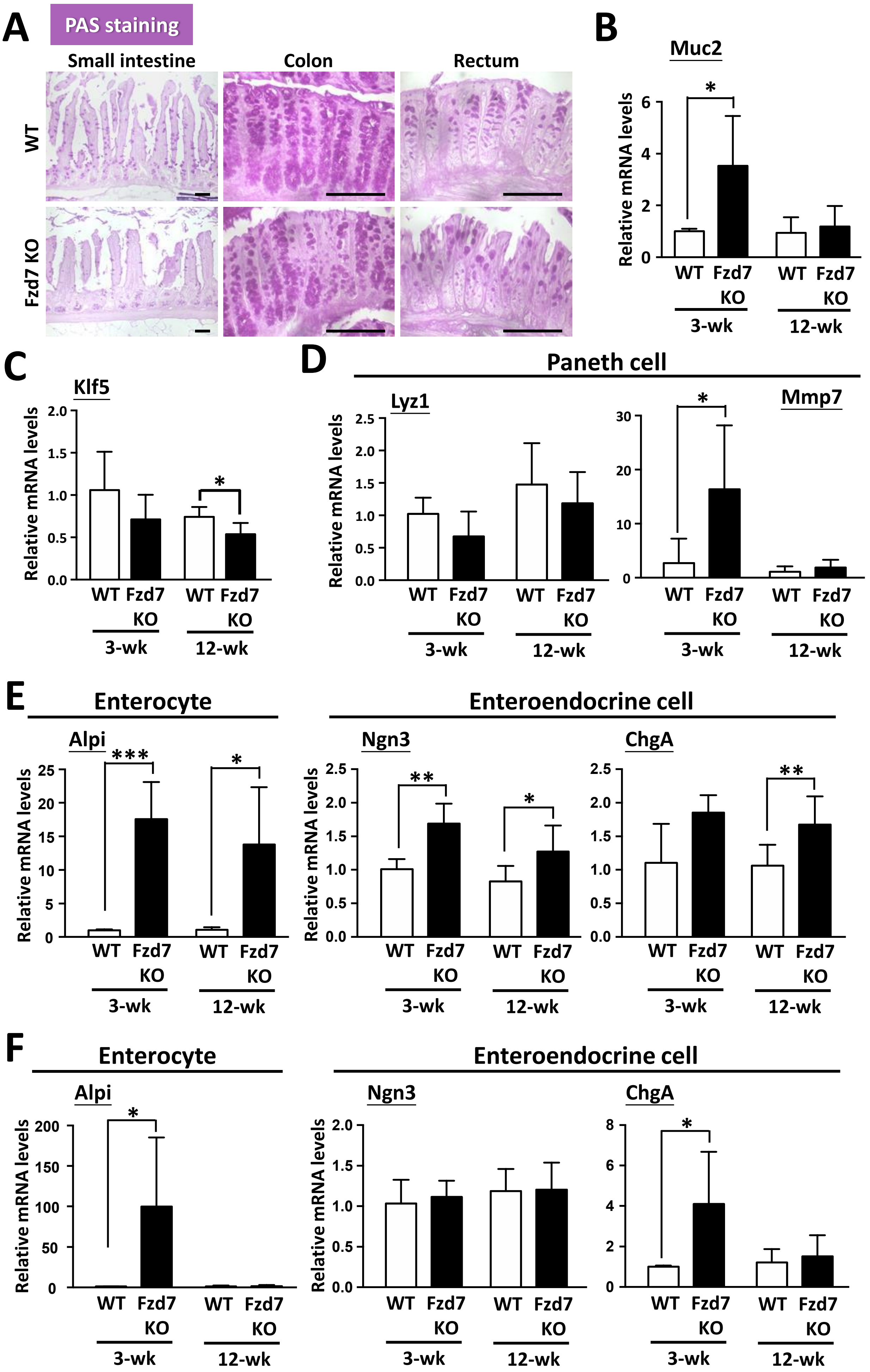

Development • Supplementary information

Supplemental Figure S3_Continued

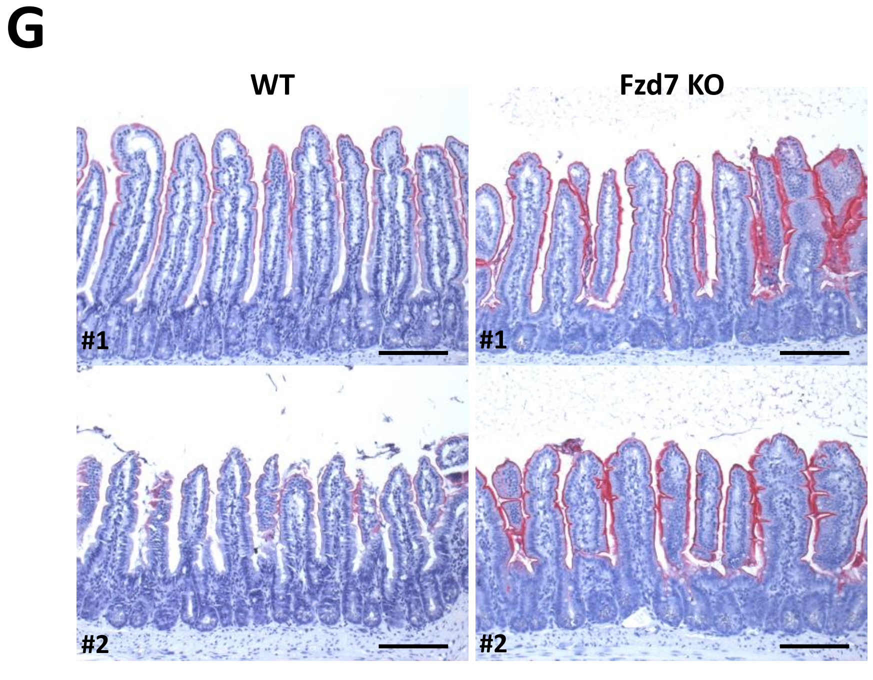

Fig. S3. Fzd7 deletion disrupted the differentiated process of intestinal epithelium. (A) PAS staining was performed on small intestine, colon and rectum of mice at 3 months of age. **(B)** The expression levels of Mus2 (goblet cell marker) was detected in colon of mice by RT-qPCR. **(C)** The expression levels of Klf5, involved in goblet cell development, was detected in small intestine of mice by RT-qPCR. **(D)** The expression levels of Lyz1 and Mmp7 (Paneth cell marker) were detected in colon of mice by RT-qPCR. **(E)** The expression levels of Alpi (enterocyte marker) and Ngn3 and ChgA (enteroendocrine cell marker) were analyzed in small intestine of mice by RT-qPCR. **(F)** The expression levels of Alpi (enterocyte marker) and Ngn3 and ChgA (enteroendocrine cell marker) were analyzed in colon of mice by RT-qPCR. **(G)** Immunohistochemistry staining of Alpi was performed on the small intestine of mice. There were 4-10 mice in each group. The results are presented as the mean ± SD. *p<0.05; **p<0.01; ***p<0.001.

Supplemental Figure S4

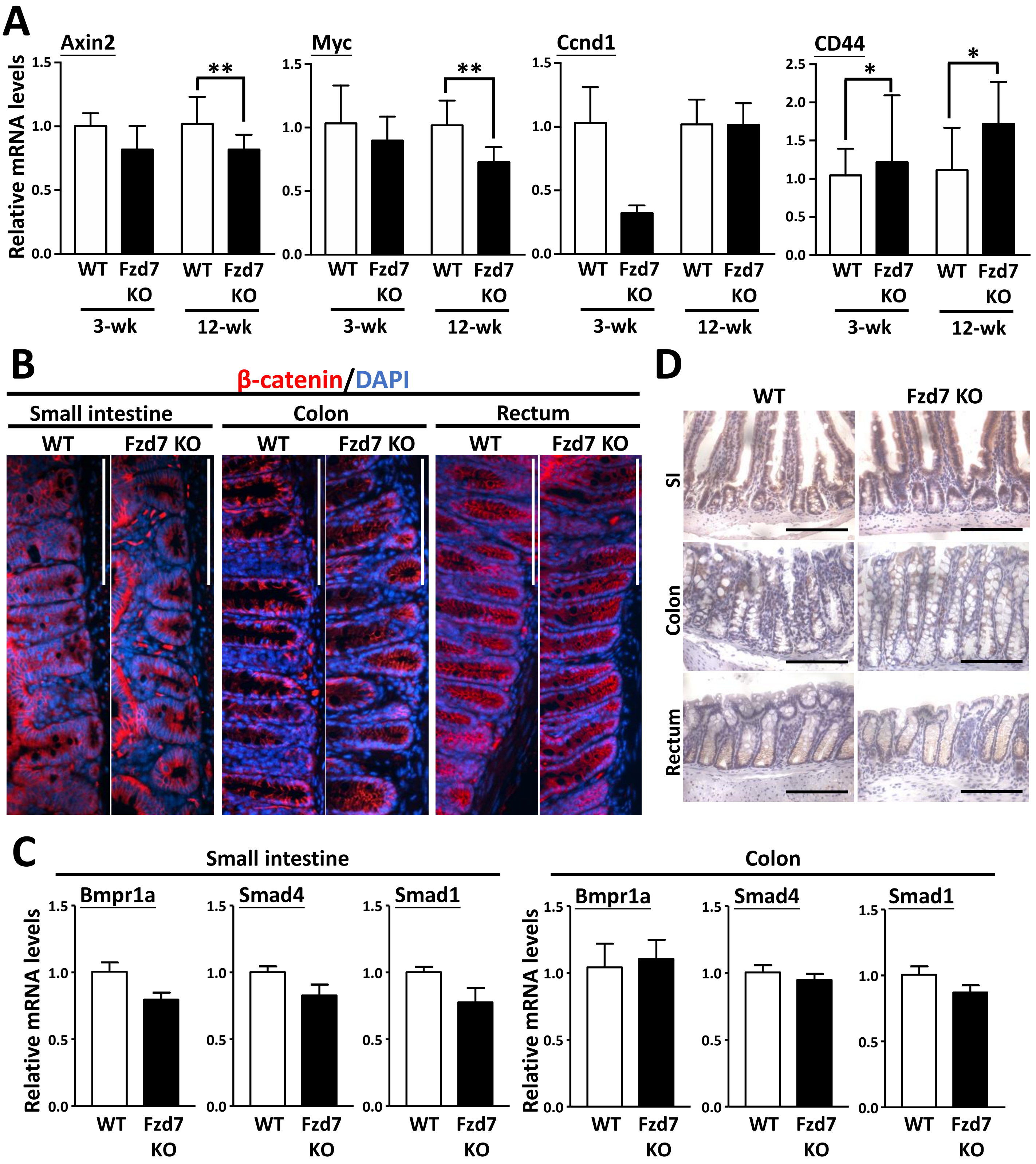

Fig. S4. The expression of genes involved in WNT canonical and BMP signaling pathway. (A) The expression changes of genes involved in WNT canonical signaling pathway were detected in colon of mice. 4-10 mice were in each group. **(B)** Immunofluorescent staining of β-catenin was analyzed in small intestine, colon and rectum of mice at 3 months of age. **(C)** The expression levels of genes involved in BMP signaling pathway were detected in mice at 3 months of age. There were four mice in each group. **(D)** Immunohistochemistry staining of Smad4 was analyzed in mice at 3 months old. The results are presented as the mean ± SD. **p*<0.05; ***p*<0.01.

Development: doi:10.1242/dev.200932: Supplementary information

Supplemental Figure S5

A

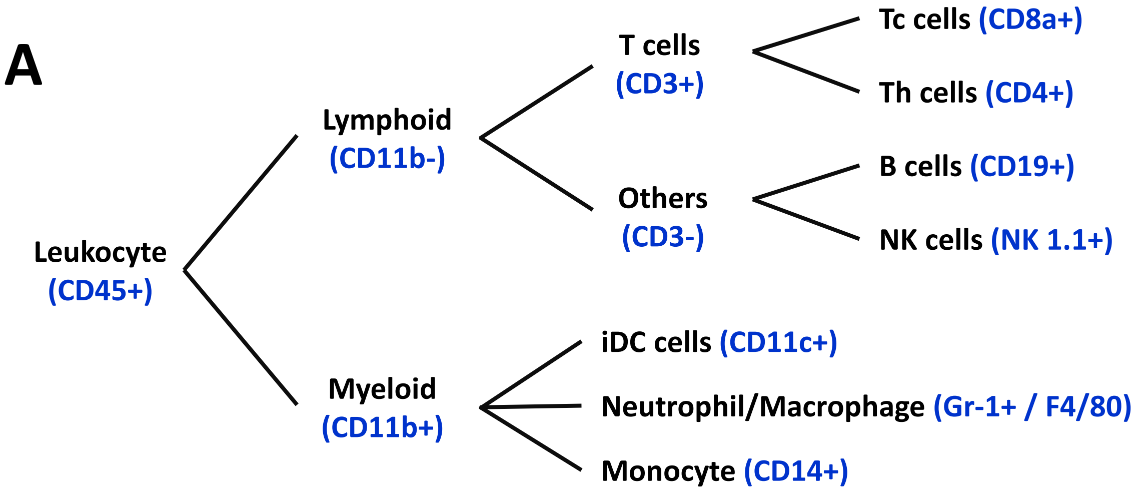

B

Assay_I

POS		NEG		BLANK	AxL 0.77	BLC (CXCL13) 0.51	CD30 Ligand (TNFSF8) 0.66	CD30 (TNFRSF8) 0.34	CD40 (TNFRSF5) 0.37	CRG-2 (CXCL10) 0.41	CTACK (CCL27) 0.50	CXCL16 0.65	Eotaxin-1 (CCL11) 0.35
*Eotaxin-2 (CCL24) 2.10	*Fas Ligand (TNFSF6) 1.79	*Fractalkine (CX3CL1) 1.80	*GCSF 1.55	GM-CSF 1.40	IFN-gamma 1.28	IGFBP-3 1.27	IGFBP-5 1.07	IGFBP-6 1.03	IL-1 alpha (IL-F1) 0.96	IL-1 beta (IL-1 F2) 0.92	IL-2 1.05	IL-3 1.06	IL-3 R beta 1.44
IL-4 1.23	*IL-5 2.11	*IL-6 1.82	*IL-9 1.73	IL-10 1.39	IL-12 p40/p70 1.19	IL-12 p70 0.99	IL-13 0.81	IL-17A 0.71	KC (CXCL-1) 0.85	Leptin R 0.92	Leptin 0.81	LIX 1.01	L-Selectin (CD62L) 1.18
Ltn (XCL1) 1.34	MCP-1 (CCL2) 1.34	*MCP-5 1.50	M-CSF 1.36	*MIG (CXCL9) 1.53	MIP-1 alpha (CCL3) 1.23	MIP-1 gamma 1.17	MIP-2 1.02	MIP-3 beta (CCL19) 0.92	MIP-3 alpha (CCL20) 0.80	PF-4 (CLCX4) 0.88	P-Selectin 0.89	RANTES (CCL5) 1.17	*SCF 1.50
SDF-1 alpha 1.14	TARC (CCL17) 1.41	I-309 (TCA-3/CCL1) 1.00	TECK (CCL25) 1.48	TIMP-1 1.32	TNF alpha 1.39	TNF RI (TNFRSF1A) 1.20	TNF RII (TNFRSF1B) 1.21	TPO 1.06	VCAM-1 (CD106) 0.91	VEFG-A 1.02	BLANK	BLANK	POS

Assay_II

POS		NEG		BLANK	bFGF 1.19	CD26 (DPPIV) 0.95	Dtk 0.71	E-Selectin 1.02	Fc gamma RIIB 1.02	Flt-3 Ligand 0.78	GITR (TNFRSF18) 1.20
*HGFR 1.53	ICAM-1 (CD54) 1.22	IGFBP-2 1.15	IGF-1 1.39	IGF-2 1.21	IL-15 1.23	IL-17 RB 1.0	IL-7 1.04	I-TAC (CXCL11) 1.08	Lungline (CXCL15) 0.92	DMC (CCL22) 0.97	MMP-2 1.23
MMP-3 1.38	OPN (SPP1) 1.23	*OPG (TNFRSF11B) 1.58	*Pro-MMP-9 1.6	Resistin 1.19	*Shh-N 1.64	TCK-1 (CXCL7) 1.21	TIMP-2 1.12	TRAMCE (TNFSF11) 1.01	TROY (TNFRSF19) 0.86	TSLP 0.85	VEFGR1 1.14
VEGFR2 1.14	VEGFR3 1.34	VEGF-D 1.21	BLANK	BLANK	BLANK	BLANK	BLANK	BLANK	BLANK	BLANK	POS

Development • Supplementary information

Supplemental Figure S5_Continued

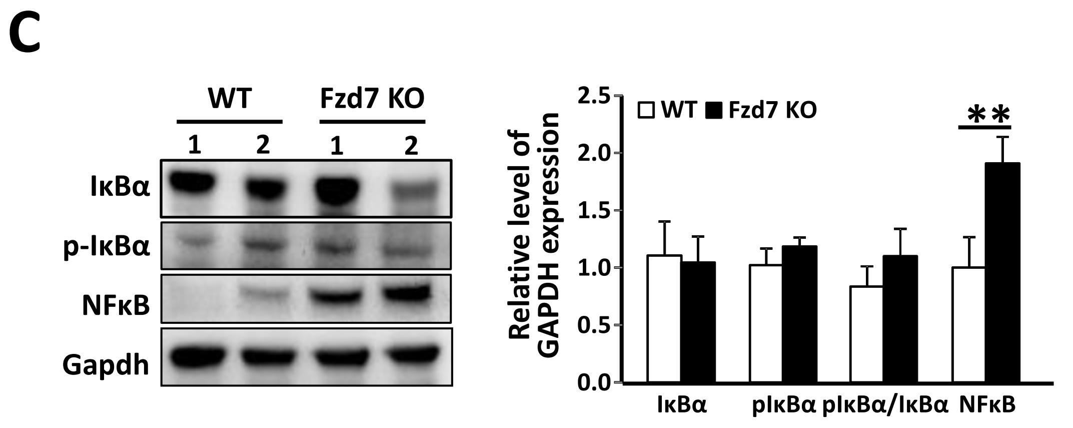

Fig. S5. Immunological responses in Fzd7 conventional knockout mice. (A) The specific markers were used for identify various types of immune cells. **(B)** Array I is the probed membrane for RayBio C-Series Mouse Cytokine Antibody Array C3 (monitoring 62 mouse proteins). Array II is the probed membrane for RayBio C-Series Mouse Cytokine Antibody Array C4(monitoring 34 mouse proteins). Red indicates the value, measured with calculated control, which was more than 1.2, means significant increase in Fzd7 deleted mice (*, value was more than 1.5). Blue indicates the value, measured with calculated control, which was less than 0.6, means significant decrease in Fzd7 deleted mice. **(C)** The protein levels in the NF-κB signaling pathway were detected in the small intestine of mice at 3 months of age. Relative protein levels were calculated using Gapdh as a calculating control. 3-4 mice were in each group. Primary antibodies: anti-NF-κB-p65 (8242, Cell Signaling Technology Inc.), anti-IκB (9242, Cell Signaling Technology Inc.), anti-p-IκB (9246, Cell Signaling Technology Inc.) The results are presented as the mean ± SD. **p<0.01.

Development • Supplementary information

Supplemental Figure S6

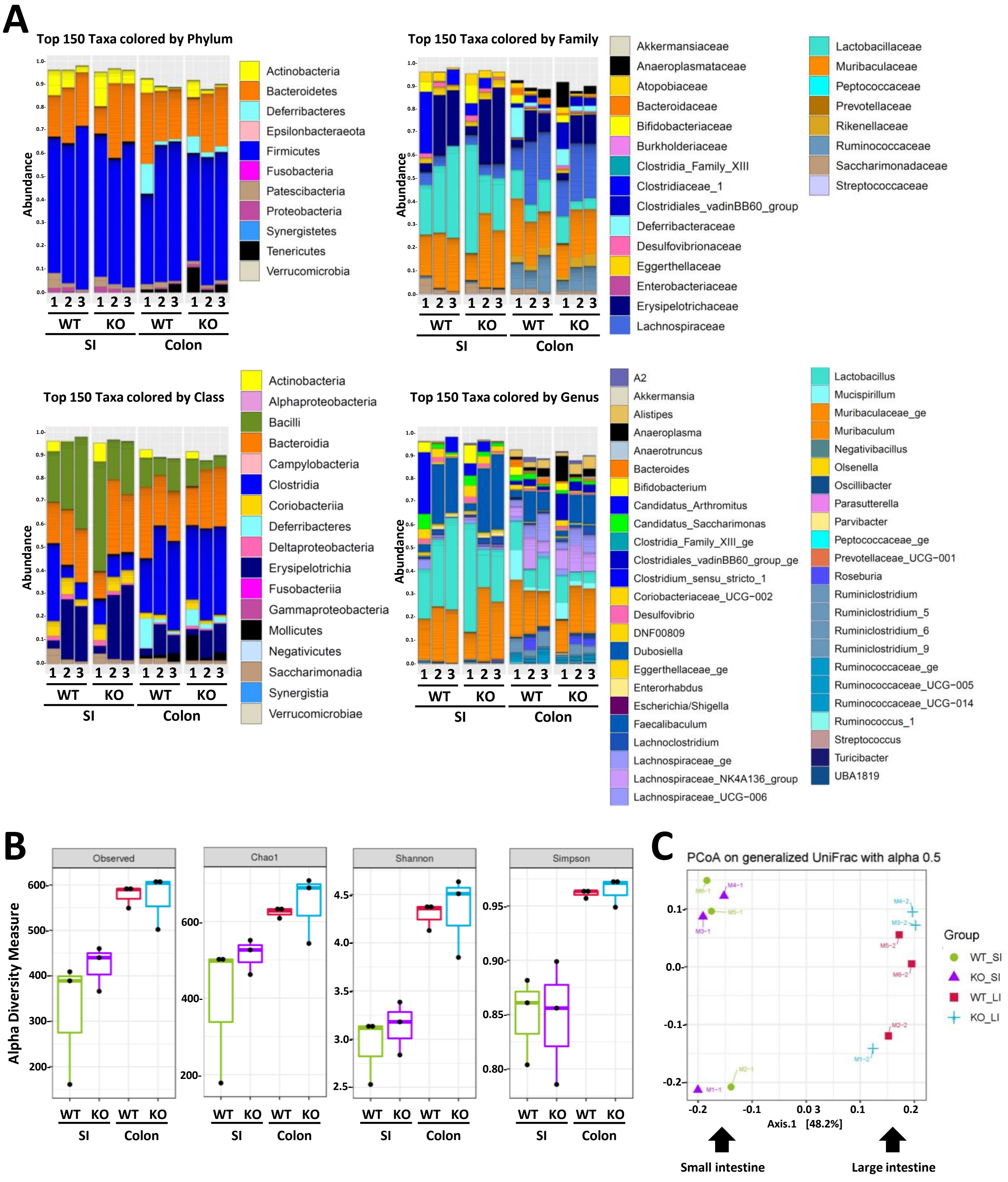

Fig. S6. Microbiota analysis of small intestine and colon in the WT and Fzd7 KO mice. (A) Relative abundance of top 150 bacterial taxa at phylum, class, family and genus level. **(B)** Alpha diversity of microbiota measured using observed species, Chao1, Shannon and Simpson diversity Index. **(C)** Beta diversity of microbiota represented by Principal Coordinates Analysis (PCoA) plot on Bray-Curtis Distance. The PCoA plots separate the small intestine from large intestine samples. Mouse age, 3 months old.

Development • Supplementary information

Supplemental Figure S7

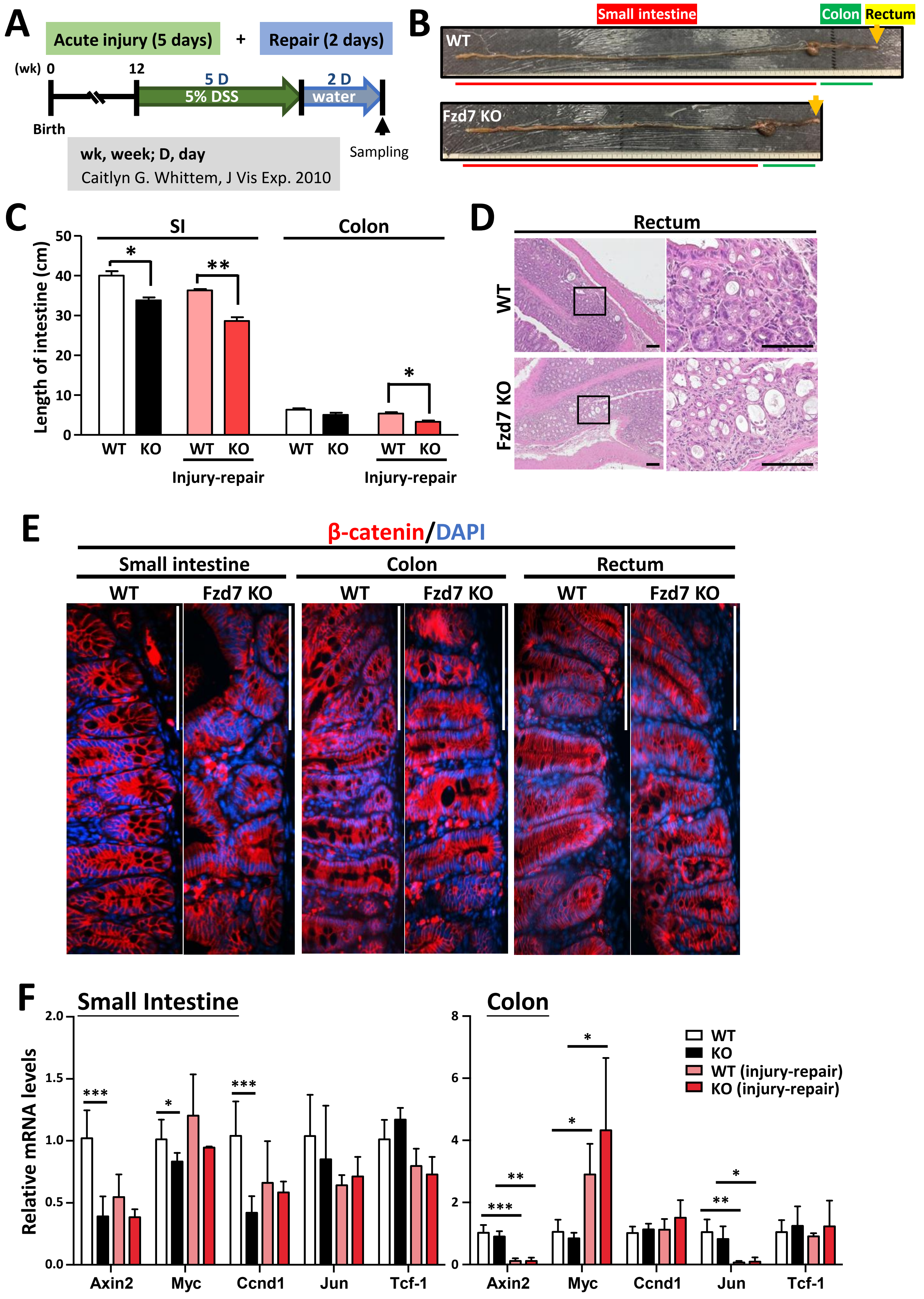

Development • Supplementary information

Fig. S7. No obvious changes of WNT signaling pathway after acute injury-repair treatment in Fzd7 knockout mice. (A) The timetable for acute injury-repair treatment. **(B)** Gross view of whole intestines in Fzd7 KO and WT mice after acute injury-repair treatment. **(C)** Quantification of length of small intestines and colon in Fzd7 KO and WT mice after acute injury-repair treatment. **(D)** More severe damage and poor repair were observed in rectum of Fzd7 KO mice after injury-repair treatment. **(E)** The immunofluorescent staining of β-catenin was performed on the small intestine, colon and rectum of mice after acute injury-repair treatment. **(F)** The genes involved in WNT signaling pathway were determined in mice with or without acute injury-repair treatment. Four mice per group were used in this figure. The results are presented as the mean ± SD. *p<0.05; **p<0.01 ; ***p<0.001.

Supplemental Figure S8

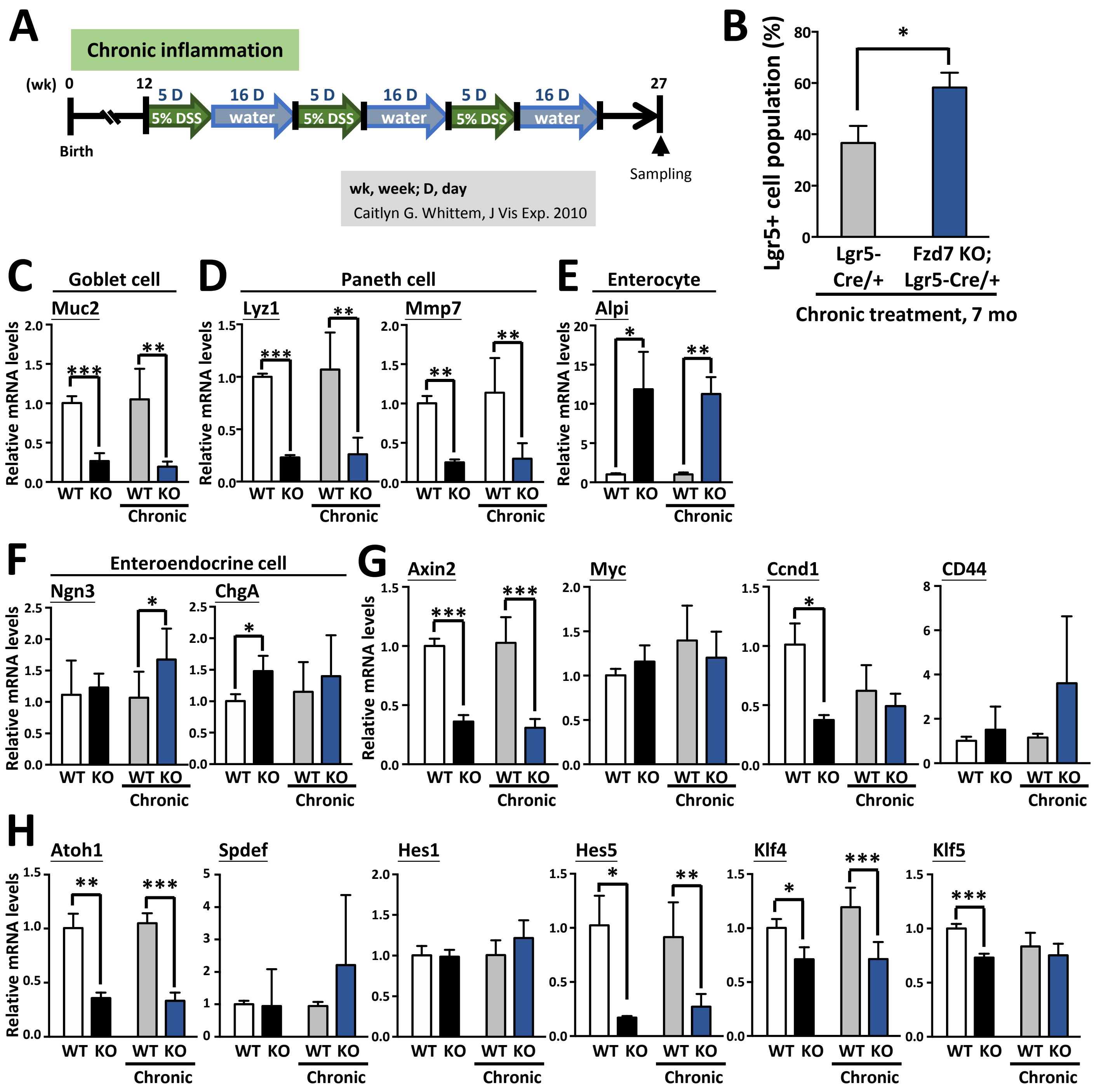

Fig. S8. The characterization of intestines in the Fzd7 deleted mice after chronic DSS injury. (A) The protocols for chronic DSS treatment on Fzd7 KO mouse models. **(B)** The EGFP indicated the Lgr5 positive cells in mice after chronic DSS injury. Quantified data was calculated via the distribution of Lgr5-positive cells versus the length of crypt. Four mice were in each group. **(C)** The goblet cell marker, Muc2, was detected in small intestine of mice with or without chronic DSS treatment. **(D)** The Paneth cell markers, Lyz1 and Mmp7, were analyzed in small intestine of mice with or without chronic DSS treatment. **(E)** The enterocyte marker, Alpi, was detected in small intestine of mice with or without chronic DSS treatment. **(F)** The enteroendocrine cell markers, Ngn3 and ChgA, were analyzed in small intestine of mice with or without chronic DSS treatment. **(G)** The mRNA expression levels of the genes involved in Wnt signaling pathway were analyzed in small intestine of mice with or without chronic DSS treatment. **(H)** The expression levels of several genes involved in goblet cell development were detected in small intestine of mice with or without chronic DSS treatment. In (C) to (H), the tissue harvested age is 7 months old and the data is measured by RT-qPCR. There were 3-8 mice in each group. The results are presented as the mean ± SD. **p*<0.05; ***p*<0.01 ; ****p*<0.001.

Development • Supplementary information

Supplemental Figure S9

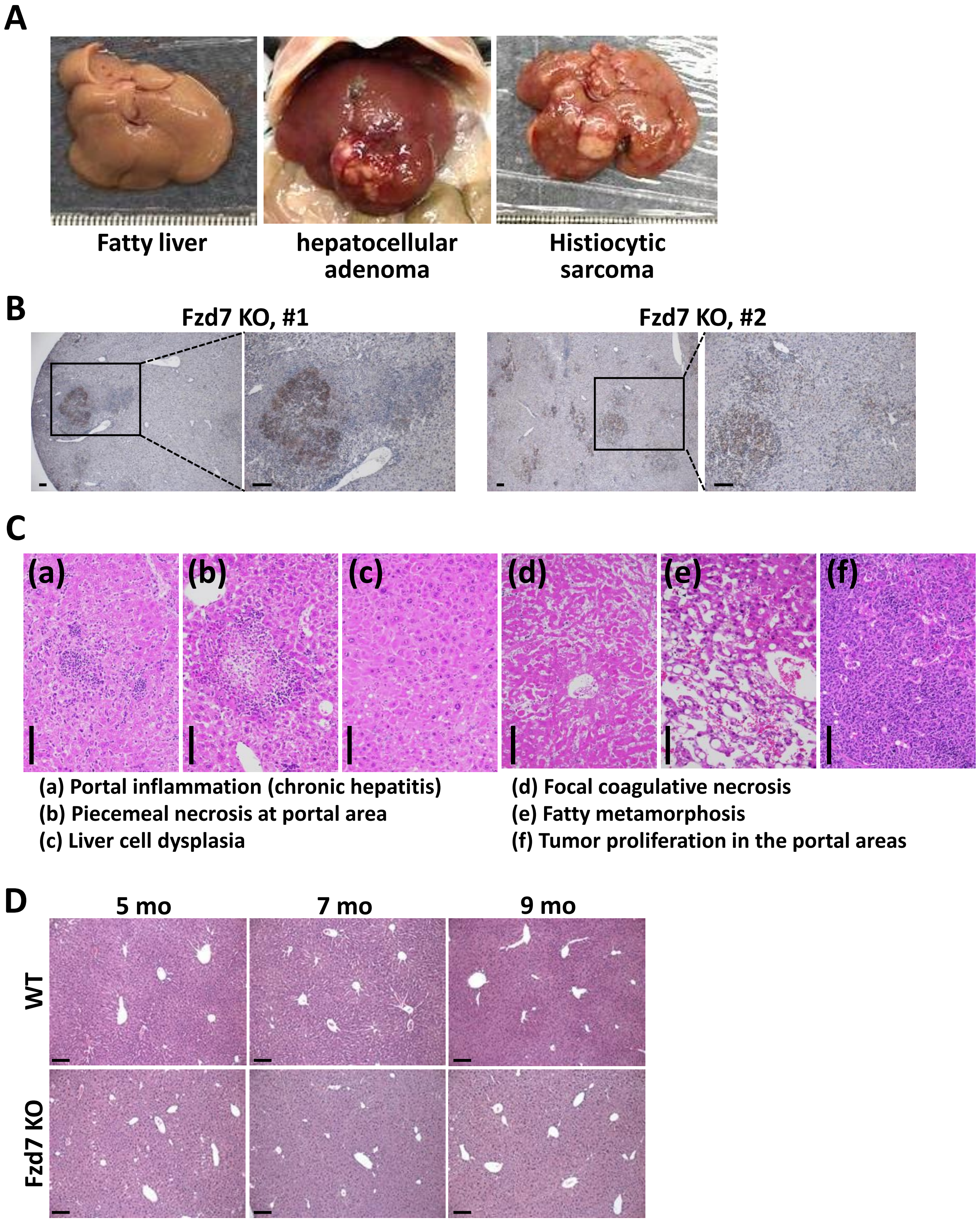

Fig. S9. Hepatocellular carcinoma of Fzd7 KO mice. (A) Gross views of whole livers harvested from Fzd7 KO mice at 12 months of age. **(B)** Histiocytic sarcoma in the liver stained with antibody F4/80 at 12 months old. **(C)** Histopathological analysis in liver of Fzd7 KO mice at 12 months old. **(D)** The H&E staining sections of liver tissue from mice at 5, 7, 9 months. Scale bar, 100µm.

Table S1. The primers and probes used for Real-time qPCR

Click here to download Table S1

Development • Supplementary information
