# Supplementary material for: Frizzled 7 modulates goblet and Paneth cell fate, and maintains homeostasis in mouse intestine
Source: Development. 2023 Feb 14;150(4):dev200932. doi: 10.1242/dev.200932 (PMC10112897; doi:10.1242/dev.200932)
Supplement: Supplementary file 1 [file develop-150-200932_review_history.pdf]

Frizzled 7 modulates goblet and Paneth cell fate, and maintains homeostasis in mouse intestine

Nai-Xin Gu, Yu-Ru Guo, Sey-En Lin, Yen-Hsin Wang, I-Hsuan Lin, Yi-Fan Chen and Yun Yen
DOI: 10.1242/dev.200932

Editor: James Wells

Review timeline

Original submission:	17 May 2022
Editorial decision:	4 July 2022
First revision received:	6 October 2022
Editorial decision:	14 November 2022
Second revision received:	16 January 2023
Accepted:	17 January 2023

Original submission

First decision letter

MS ID#: DEVELOP/2022/200932

MS TITLE: Frizzled-7 modulates Goblet and Paneth cell fate and maintains the homeostasis in intestines

AUTHORS: Nai-Xin Gu, Yu-Ru Guo, Sey-En Lin, Yen-Hsin Wang, I-Hsuan Lin, Yun Yen, and Yi-Fan Chen

I have now received all the referees' reports on the above manuscript, and have reached a decision. The referees' comments are appended below, or you can access them online: please go to BenchPress and click on the 'Manuscripts with Decisions' queue in the Author Area.

As you will see, the referees express considerable interest in your work, but have some significant criticisms and recommend a substantial revision of your manuscript before we can consider publication. If you are able to revise the manuscript along the lines suggested, which may involve further experiments, I will be happy receive a revised version of the manuscript. Your revised paper will be re-reviewed by one or more of the original referees, and acceptance of your manuscript will depend on your addressing satisfactorily the reviewers' major concerns. Please also note that Development will normally permit only one round of major revision. If it would be helpful, you are welcome to contact us to discuss your revision in greater detail. Please send us a point-by-point response indicating your plans for addressing the referee’s comments, and we will look over this and provide further guidance.

Please attend to all of the reviewers' comments and ensure that you clearly highlight all changes made in the revised manuscript. Please avoid using 'Tracked changes' in Word files as these are lost in PDF conversion. I should be grateful if you would also provide a point-by-point response detailing how you have dealt with the points raised by the reviewers in the 'Response to Reviewers' box. If you do not agree with any of their criticisms or suggestions please explain clearly why this is so.

© 2023. Published by The Company of Biologists under the terms of the Creative Commons Attribution License (https://creativecommons.org/licenses/by/4.0/).

Reviewer 1

Advance summary and potential significance to field

In the manuscript entitled “Frizzled-7 modulates Goblet and Paneth cell fate and maintains the homeostasis in intestines” Gu et al analyse the intestinal phenotype of conventional Fzd7 KO mice, where they show that intestinal homeostasis is lost, resulting in shorter crypts and small intestine length decrease, as well as barrier impairment leading to microbiota imbalance and an overall inflammatory response. They also show that loss of Fzd7 acts synergistically with chemical-induced colitis, delaying recovery and in some cases inducing tumorigenesis. In all, this study sheds light into the complex interplay between Wnt and Notch signalling pathways in maintaining intestinal homeostasis and the importance of the microenvironment in tumour progression.
The presented data is of good quality and broadly supports the claims of the authors.

Comments for the author

Overall this is an interesting study that contrasts previous reports where an enterocolitis phenotype was not observed in Fzd7 KO specifically in Lgr5+ cells.
The results of both reports are not necessarily contradictory, but rather could be explained by the complex interplay between the many types of cells residing at the bottom of the crypts (and in their surroundings), in addition to the complex interplay between Wnt and Notch signalling pathways, especially in ISCs.

I would suggest that the authors discuss a bit more about how Fzd7 expression in other cells rather than only on Lgr5+ cells could result in the described phenotypes.
Moreover, I would suggest Hes1 expression going up to be shown on the main figure alongside Atoh1 expression going down. It has been shown that Hes1 downstream of active Notch signalling inhibits Atoh1 expression in progenitor cells, while Atoh1 expressing cells become secretory progenitors. Maybe performing a double active notch 1 (intracellular) / beta-catenin staining in the Fzd7 KO background could help clarify whether the simultaneous co-activation of both pathways is affected in this specific context, and explain how some of the phenotypes resemble a Notch-gain-of-function phenotype (loss of secretory cells, increase on absorptive cells).

To delve into the role of NF-kB signalling being affected by Fzd7 deletion and its potential link to the inflammatory phenotype, gene expression could be analysed by qPCR of targets such us IL-1, IL-2 or IFNg in intestinal cell extracts. Alternatively, general NF-kB activity could be assessed by Western Blot of NF-kB p65, phospho-NF-kB p65 (Ser468), and IkBa on intestinal cell extracts of wildtype vs. Fzd7-KO mice.

Minor comments:

Please specify how many crypts or crypt/villi units where quantified for IHC/IF cell quantifications instead of “slides”. The authors should also specify whether cell countings where performed in similar or comparable small intestinal regions, as the number of Goblet Cells, for instance, varies greatly along the small intestine.
On another a minor point, it would be ideal to have single channel images beta-catenin IF data. It is hard to make out nuclear areas with the images provided.
Gene names could be italicized.

Reviewer 2

Advance summary and potential significance to field

Gut et al., analyzed Fzd7 KO mice and found alterations in both the number of stem cells and their differentiation. Utilizing the DSS colitis model, they also showed that these mice promote colitis. The role of Fzd7 in the intestine was previously described (PMID 25892522). The authors found that Fzd7 KO mice exhibit rectal adenocarcinomas (when combined with chronic DSS injury) and tumors in the liver. While this new finding is interesting, their analysis is still preliminary. In addition, some of their claims were not experimentally supported.

© 2023. Published by The Company of Biologists under the terms of the Creative Commons Attribution License (https://creativecommons.org/licenses/by/4.0/).

Comments for the author

A previous study (PMID 25892522) analyzed epithelial conditional Fzd7 KO mice using the Lgr5-CreERT allele and AhCre allele (targeting all epithelial cells except Paneth Cells), and showed impaired stem cell activity upon Fzd7 deletion. In contrast, the authors showed that the number of stem cells increases upon Fzd7 KO (Fig 1H). This discrepancy should be discussed, and the authors should analyze other stem cell markers such as OLFM4 (qPCR assays and IHC/IF analysis).

The same study mentioned above also analyzed conventional Fzd7 KO mice and showed that these mice exhibit impaired regeneration upon irradiation. Surprisingly, the authors failed to discuss this highly relevant work. Would this regeneration defect and the authors' DSS phenotypes still imply a role for Fzd7 in epithelial cells? Could it instead play a role in immune cells?

The authors showed that Wnt signaling is disrupted upon Fzd7 KO (Fig 2J), but they stated, "Consequently, beta-catenin was activated and entered the nucleus of intestinal epithelial cells in Fzd7 KO mice (Supplemental Figure S4B)." This interpretation makes no sense, nor is the data convincing.

The authors claimed dysregulation of Notch signals, but only Hes5 was analyzed. How about Hes1 expression?

The authors claimed that a few Fzd KO mice with chronic DSS injury developed rectal adenocarcinoma (Fig 5G). However, the histological analysis is very limited, and no quantification was provided.

The authors claimed that Fzd7 deletion induces liver tumorigenesis (Fig 6E), but its characterization seems extremely preliminary, lacking both histology and quantification. Is Fzd7 expressed in the liver?

The microbiome analysis (Fig 6-D) seems unclear. Any changes after 12 months would likely reflect secondary effects due to the goblet cell differentiation defects. Unless they show some functional evidence of changed gut microbiota, the data isn't informative.

First revision

<u>Author response to reviewers' comments</u>

Reviewer 1 Advance Summary and Potential Significance to Field:
In the manuscript entitled "Frizzled-7 modulates Goblet and Paneth cell fate and maintains the homeostasis in intestines" Gu et al analyse the intestinal phenotype of conventional Fzd7 KO mice, where they show that intestinal homeostasis is lost, resulting in shorter crypts and small intestine length decrease, as well as barrier impairment leading to microbiota imbalance and an overall inflammatory response. They also show that loss of Fzd7 acts synergistically with chemical-induced colitis, delaying recovery and in some cases inducing tumorigenesis. In all, this study sheds light into the complex interplay between Wnt and Notch signalling pathways in maintaining intestinal homeostasis and the importance of the microenvironment in tumour progression. The presented data is of good quality and broadly supports the claims of the authors.

Reviewer 1 Comments for the Author:

1. Overall this is an interesting study that contrasts previous reports where an enterocolitis phenotype was not observed in Fzd7 KO specifically in Lgr5+ cells. The results of both reports are not necessarily contradictory, but rather could be explained by the complex interplay between the many types of cells residing at the bottom of the crypts (and in their surroundings), in addition to the complex interplay between Wnt and Notch signalling pathways, especially in ISCs. I would suggest that the authors discuss a bit more about how Fzd7 expression in other cells rather than only on Lgr5+ cells could result in the described phenotypes.

© 2023. Published by The Company of Biologists under the terms of the Creative Commons Attribution License (https://creativecommons.org/licenses/by/4.0/).

Response:
Thanks for your valuable suggestion. In the section of Discussion, we added the discussion of this comment as below.
(Line 296-301) "Enterocolitis is induced by an imbalance between proinflammatory and anti-inflammatory processes, disturbed epithelial homeostasis and a changed distribution of the microbiota. We used a conventional knockout model to confirm that Fzd7 deficiency causes severe enterocolitis in mice. As our data shown, both isolated intestinal epithelium and circulated immune cells had Fzd7 expression; therefore, Fzd7 deletion may have effects on other intestinal epithelial cells other than stem cells."

2. Moreover, I would suggest Hes1 expression going up to be shown on the main figure alongside Atoh1 expression going down. It has been shown that Hes1 downstream of active Notch signalling inhibits Atoh1 expression in progenitor cells, while Atoh1 expressing cells become secretory progenitors. Maybe performing a double active notch 1 (intracellular) / beta-catenin staining in the Fzd7 KO background could help clarify whether the simultaneous co-activation of both pathways is affected in this specific context, and explain how some of the phenotypes resemble a Notch-gain-of-function phenotype (loss of secretory cells, increase on absorptive cells).

Response:
Thank you for your suggestions. We added the new data in Figure 2 and the signaling regulation in Figure 7 for explaining the regulation of secretory and absorptive cells. As our data shown, Fzd7 deletion led to the imbalanced distribution of epithelial cells resulting in a decreased ratio of secretory/absorptive cells. Atoh1 was dominantly downregulated through Wnt signaling (low expression of beta-catenin in nucleus) in Fzd7 KO mice; meanwhile upregulated Notch signaling increased Hes1 expression. These data were added in Figure 2 (F)(K)(L) and the signaling regulation was added in Figure 7.

3. To delve into the role of NF-kB signalling being affected by Fzd7 deletion and its potential link to the inflammatory phenotype, gene expression could be analysed by qPCR of targets such us IL-1, IL-2 or IFNg in intestinal cell extracts. Alternatively, general NF-kB activity could be assessed by Western Blot of NF-kB p65, phospho-NF-kB p65 (Ser468), and IkBa on intestinal cell extracts of wildtype vs. Fzd7-KO mice.

Response:
Thank you for your valuable suggestions. We analyzed the NF-κB signaling expression which was upregulated in Fzd7 KO mice (Supplemental Figure S5C). We also re-confirmed the expression of key proinflammatory cytokines, TNFα and IL-6, and they were upregulated in Fzd7 KO mice (Figure 3D-E). The protein expression of proinflammatory cytokines may directly demonstrate the inflammatory phenotypes in Fzd7 deleted condition.

4. Minor comments: Please specify how many crypts or crypt/villi units where quantified for IHC/IF cell quantifications instead of "slides".

Response:
Thanks for your suggestions. We modified and carefully described the calculation in the Figure Legends as below.
(Line 783-785) "(E) Quantified data of crypt length in the small intestines of mice. The crypt lengths are expressed as mean length, measurements were taken from at least 9 crypts for each sample. Three mice were in each group."
(Line 785-795) "(G) Quantified data for migrated EdU+ cells in the small intestines of mice. Migration distance was defined as the distance from villus bottom to foremost EdU positive cell. Measurements were taken from average 10 villus were measured for each sample. Three mice were in each group. (H) Tracing of Lgr5-expressed cells (GFP+ cells) in the small intestine, colon, and rectum of Fzd7-KO mice with Lgr5-EGFP-Cre transgene at 3 months of age. The distribution of Lgr5+ cells in the small intestine of mice at 3 months of age. Quantified data was calculated via the distribution of Lgr5-positive cells versus the length of crypt. Measurements were taken from at average 7 crypts for each sample. Three mice were in each group."
(Line 800-802) "(B) Quantitative data of goblet cell numbers in the intestinal sections. Measurements were taken from average 40 villus for each sample. WT groups had four mice at 3 weeks of age and three mice at 12 weeks of age; six mice were in both Fzd7 KO groups."
(Line 844-847) "(E) Quantification of goblet cell numbers in intestines after acute injury-

© 2023. Published by The Company of Biologists under the terms of the Creative Commons Attribution License (https://creativecommons.org/licenses/by/4.0/).

repair treatment. Except non-treated Fzd7 KO group (n=6), the other groups had three mice. Measurements were taken from average 40 villus for each sample."

5. The authors should also specify whether cell countings where performed in similar or comparable small intestinal regions, as the number of Goblet Cells, for instance, varies greatly along the small intestine.

Response:

Thanks for your suggestions. The intestinal tissues were obtained by the following step. The front end and rear end which account for 1/9 of the total length were removed. The imaging of the intestine was mainly from middle part to distal part, which are approximately equivalent to jejunum and ileum. We added this description in Materials and Methods, Line 407-409.

6. On another a minor point, it would be ideal to have single channel images beta- catenin IF data. It is hard to make out nuclear areas with the images provided.

Response:

Thank you for your comment. We enlarged the image and calculated the ratio in the entry of beta-catenin in the Fzd7 KO mice in Figure 2.

7. Gene names could be italicized.

Response:

Thank you for your comment. We modified all the gene names in the manuscript.

Reviewer 2 Advance Summary and Potential Significance to Field:

Gut et al., analyzed Fzd7 KO mice and found alterations in both the number of stem cells and their differentiation. Utilizing the DSS colitis model, they also showed that these mice promote colitis. The role of Fzd7 in the intestine was previously described (PMID 25892522). The authors found that Fzd7 KO mice exhibit rectal adenocarcinomas (when combined with chronic DSS injury) and tumors in the liver. While this new finding is interesting, their analysis is still preliminary. In addition, some of their claims were not experimentally supported.

Reviewer 2 Comments for the Author:

1. A previous study (PMID 25892522) analyzed epithelial conditional Fzd7 KO mice using the Lgr5-CreERT allele and AhCre allele (targeting all epithelial cells except Paneth Cells), and showed impaired stem cell activity upon Fzd7 deletion. In contrast, the authors showed that the number of stem cells increases upon Fzd7 KO (Fig 1H). This discrepancy should be discussed, and the authors should analyze other stem cell markers such as OLFM4 (qPCR assays and IHC/IF analysis).

Response:

Thank you for your valuable comment. We analyzed another intestinal stem cell marker, Olfm4, using qPCR assays and IF analysis, but there was no obvious alteration between the Fzd7 KO and WT mice. In the article (PMID 25892522), Fzd7 was deleted at 6-12 weeks old. The AhCre, which is driven by a cytochrome P450 promoter element, could be expressed in liver, intestine, pancreas, gallbladder, esophagus, and stomach (Ireland et al., 2004). Because we didn't systemically analyze and compare these two mouse models, we cannot explain the critical reason for their own different phenotypes. In our model, we posited that Fzd7 deletion may disturb the cell differentiation of the intestinal epithelium, resulting in feedback modulating the proliferation of intestinal stem cells. Our data revealed that loss of Fzd7 along the crypt-villus axis slowed cell migration and interrupted crypt extension but had no noticeable effects on villus extension. (These were described on Line 133-138).

<u>Reference</u>: Ireland, H., Kemp, R., Houghton, C., Howard, L., Clarke, A.R., Sansom, O.J., and Winton, D.J. (2004). Inducible Cre-mediated control of gene expression in the murine gastrointestinal tract: effect of loss of beta-catenin. Gastroenterology 126, 1236-1246.

2. The same study mentioned above also analyzed conventional Fzd7 KO mice and showed that these mice exhibit impaired regeneration upon irradiation. Surprisingly, the authors failed to discuss this highly relevant work. Would this regeneration defect and the authors' DSS phenotypes still imply a role for Fzd7 in epithelial cells? Could it instead play a role in immune cells?

© 2023. Published by The Company of Biologists under the terms of the Creative Commons Attribution License (https://creativecommons.org/licenses/by/4.0/).

Response:
Thank you for your comment and suggestions. Both of irradiation and DSS treatment can damage intestinal epithelium and induce inflammation. Indeed, in the article (PMID 25892522), they analyzed the PCNA for indicating the proliferating cells, but they did not demonstrate that those cells were stem cells or other cells under proliferation. In our study, we found that damages, such as genetic, environmental, or aging effects, could interrupt the homeostasis and functions of intestinal stem cells. For example, age effects could enhance stem cell proliferation and interfere stem cell differentiation. Fzd7 were expressed in PBMC of circulation and deleted in our knockout mouse models. Therefore, in future, we will analyze the details of Fzd7 function in immune cells.

3. The authors showed that Wnt signaling is disrupted upon Fzd7 KO (Fig 2J), but they stated, “Consequently, beta-catenin was activated and entered the nucleus of intestinal epithelial cells in Fzd7 KO mice (Supplemental Figure S4B).” This interpretation makes no sense, nor is the data convincing.

Response:
Thank you for your correction. We corrected this sentence as below.
“Consequently, beta-catenin was not activated and entered the nucleus of intestinal epithelial cells in Fzd7 KO mice (Supplemental Figure S4B).”

4. The authors claimed dysregulation of Notch signals, but only Hes5 was analyzed. How about Hes1 expression?

Response:
Thank you for your suggestions. We added the new data in Figure 2 and the signaling regulation in Figure 7 for explaining the regulation of secretory and absorptive cells. As our data shown, Fzd7 deletion led to the imbalanced distribution of epithelial cells resulting in a decreased ratio of secretory/absorptive cells. Atoh1 was dominantly downregulated through Wnt signaling (low expression of beta-catenin in nucleus) in Fzd7 KO mice; meanwhile upregulated Notch signaling increased Hes1 expression. These data were added in Figure 2 (F)(K)(L) and the signaling regulation was added in Figure 7.

5. The authors claimed that a few Fzd KO mice with chronic DSS injury developed rectal adenocarcinoma (Fig 5G). However, the histological analysis is very limited, and no quantification was provided.

Response:
Thank you for your comments. In this part, we analyzed eight Fzd7 knockout mice and one of them had severe rectal adenocarcinoma; however, other mice had few but not obvious histological pattern of rectal adenocarcinoma. We modified the description in results as below.
(Line 240-241) “A Fzd7 KO mouse with chronic DSS injury developed rectal adenocarcinoma (1/8= 12.5%) (Fig. 5G).”

6. The authors claimed that Fzd7 deletion induces liver tumorigenesis (Fig 6E), but its characterization
seems extremely preliminary, lacking both histology and quantification. Is Fzd7 expressed in the liver?

Response:
Thank you for your comment. Fzd7 is expressed in the liver of WT mice and also deleted in the liver of Fzd7 KO mice. When we analyzed the histopathology of livers at 5, 7 and 9 months old (Figure S9C), the results were no obvious defects in Fzd7 deleted model. As other articles reported, the defects in intestines could induce the pathological phenomenon in liver through the imbalance of intestinal microbiota. Therefore, we pursued Fzd7 deletion led the primary defects on intestines (around 3 months old) and then induced liver pathogenesis (around 12 months old).

7. The microbiome analysis (Fig 6-D) seems unclear. Any changes after 12 months would likely reflect secondary effects due to the goblet cell differentiation defects. Unless they show some functional evidence of changed gut microbiota, the data isn’t informative.

© 2023. Published by The Company of Biologists under the terms of the Creative Commons Attribution License (https://creativecommons.org/licenses/by/4.0/).

Response:
Thank you for your comment. As we described previously, in this Fzd7 knockout mouse model, the primary defects could occur on intestinal epithelium, similar to DSS treatment. The impaired and imbalanced epithelium exhausted the whole intestines and also destroyed the homeostasis of intestinal microbiota. However, the distribution and types of microbiota contribute to the health of intestines and the metabolites produced by microbiota can damage other tissues and organs. In the future, we continue to study these connections between intestines and other organs.

Second decision letter

MS ID#: DEVELOP/2022/200932

MS TITLE: Frizzled-7 modulates Goblet and Paneth cell fate and maintains the homeostasis in intestines

AUTHORS: Nai-Xin Gu, Yu-Ru Guo, Sey-En Lin, Yen-Hsin Wang, I-Hsuan Lin, Yun Yen, and Yi-Fan Chen

I have now received all the referees reports on the above manuscript, and have reached a decision. The referees' comments are appended below, or you can access them online: please go to BenchPress and click on the 'Manuscripts with Decisions' queue in the Author Area.

The overall evaluation is positive and we would like to publish a revised manuscript in Development, provided that the referees' comments can be satisfactorily addressed. One referee still had some concerns about data interpretation that I think can be addressed by revising the text. For example, I agree that 1/8 animals with rectal adenocarcinoma is not likely significant and the manuscript should clearly indicate this. Also, the discussion should address possible reasons why phenotypes in this report differ from those previously published. However, I do not think that any additional experiments need to be performed. Please attend to this reviewers comments in your revised manuscript and detail them in your point-by-point response. If you do not agree with any of their criticisms or suggestions, explain clearly why this is so. If it would be helpful, you are welcome to contact us to discuss your revision in greater detail. Please send us a point-by-point response indicating your plans for addressing the referee’s comments, and we will look over this and provide further guidance.

Reviewer 1

Advance summary and potential significance to field

In the manuscript entitled “Frizzled-7 modulates Goblet and Paneth cell fate and maintains the homeostasis in intestines” Gu et al analyse the intestinal phenotype of conventional Fzd7 KO mice, where they show that intestinal homeostasis is lost, resulting in shorter crypts and small intestine length decrease, as well as barrier impairment leading to microbiota imbalance and an overall inflammatory response. They also show that loss of Fzd7 acts synergistically with chemical-induced colitis, delaying recovery and in some cases inducing tumorigenesis. In all, this study sheds light into the complex interplay between Wnt and Notch signalling pathways in maintaining intestinal homeostasis and the importance of the microenvironment in tumour progression.
The presented data is of good quality and broadly supports the claims of the authors.

Comments for the author

Authors have addressed most raised issues and adapted the manuscript accordingly.
The quality of the manuscript has been improved by adding insight into the molecular mechanism of possible homeostasis impairment in intestinal epithelial cells (Atoh1 and Hes1 misregulation), and involvement of NF-kB signaling in the disruption of the inflammatory status. I thank the authors for performing these informative experiments, improving the methods and figure legends, as well

© 2023. Published by The Company of Biologists under the terms of the Creative Commons Attribution License (https://creativecommons.org/licenses/by/4.0/).

as improving the pictures. Although I believe that nuclear beta-catening staining (and lack thereof) could still be more clear (either by improving the confocal microscopy images, showing a greater magnification, or even using an specific active-beta-catenin antibody [05-665 Sigma-Aldrich]) I recommend to accept the paper in its current form.

Reviewer 2

Advance summary and potential significance to field

While a previous study (PMID 25892522) has already analyzed both conditional and whole body Fzd7 KO mice, but described very different gut phenotypes from those found in the authors' KO mice. To improve the impact of the current study, the authors should further investigate the mechanisms underlying this discrepancy.

Comments for the author

The published work (PMID 25892522) has already analyzed both conditional and whole body Fzd7 KO mice but these mice had distinct gut phenotypes from those found in the authors' KO mice. While the authors have addressed some aspects in their response letter, they still failed to discuss them in the revised manuscript.
Given this earlier publication showing the conflicting data, the authors should perform additional studies to clarify the discrepancy, providing a clear explanation.

Many revised sentences in the text are unclear and require editing:

For example, line 163-164: "Consequently, beta-catenin was not activated and entered the nucleus of intestinal epithelial cells in Fzd7 KO mice (Supplemental Figure S4B)."

The authors claimed Fzd KO mice with chronic DSS injury developed rectal adenocarcinoma (main Fig 5G), but they stated that only 1 out of 8 mice had this phenotype. In order to claim rectal adenocarcinoma, they should increase sample size.

What is the sample size for liver tumor analysis of 12 month-old mice? What percentage of mice developed liver tumors? In addition, the authors still claimed that alterations in intestinal microbiota could induce tumorigenesis (line 264-266), but no functionally supporting data was provided.

Second revision

Author response to reviewers' comments

Reviewer 1 Advance summary and potential significance to field

Reviewer 1 Advance Summary and Potential Significance to Field:
In the manuscript entitled "Frizzled-7 modulates Goblet and Paneth cell fate and maintains the homeostasis in intestines" Gu et al analyse the intestinal phenotype of conventional Fzd7 KO mice, where they show that intestinal homeostasis is lost, resulting in shorter crypts and small intestine length decrease, as well as barrier impairment leading to microbiota imbalance and an overall inflammatory response. They also show that loss of Fzd7 acts synergistically with chemical-induced colitis, delaying recovery and in some cases inducing tumorigenesis. In all, this study sheds light into the complex interplay between Wnt and Notch signalling pathways in maintaining intestinal homeostasis and the importance of the microenvironment in tumour progression. The presented data is of good quality and broadly supports the claims of the authors.

© 2023. Published by The Company of Biologists under the terms of the Creative Commons Attribution License (https://creativecommons.org/licenses/by/4.0/).

Reviewer 1 Comments for the Author:
Authors have addressed most raised issues and adapted the manuscript accordingly. The quality of the manuscript has been improved by adding insight into the molecular mechanism of possible homeostasis impairment in intestinal epithelial cells (Atoh1 and Hes1 misregulation), and involvement of NF-kB signaling in the disruption of the inflammatory status. I thank the authors for performing these informative experiments, improving the methods and figure legends, as well as improving the pictures. Although I believe that nuclear beta-catenin staining (and lack thereof) could still be more clear (either by improving the confocal microscopy images, showing a greater magnification, or even using an specific active-beta-catenin antibody [05-665 Sigma-Aldrich]) I recommend to accept the paper in its current form.

Response

Thank you for your valuable comment. We ordered this antibody (active-beta-catenin antibody [05-665 Sigma-Aldrich]) and did the experiment again. However, in our protocol and model, we cannot do the better result using the active-β-catenin antibody [05-665 Sigma-Aldrich].

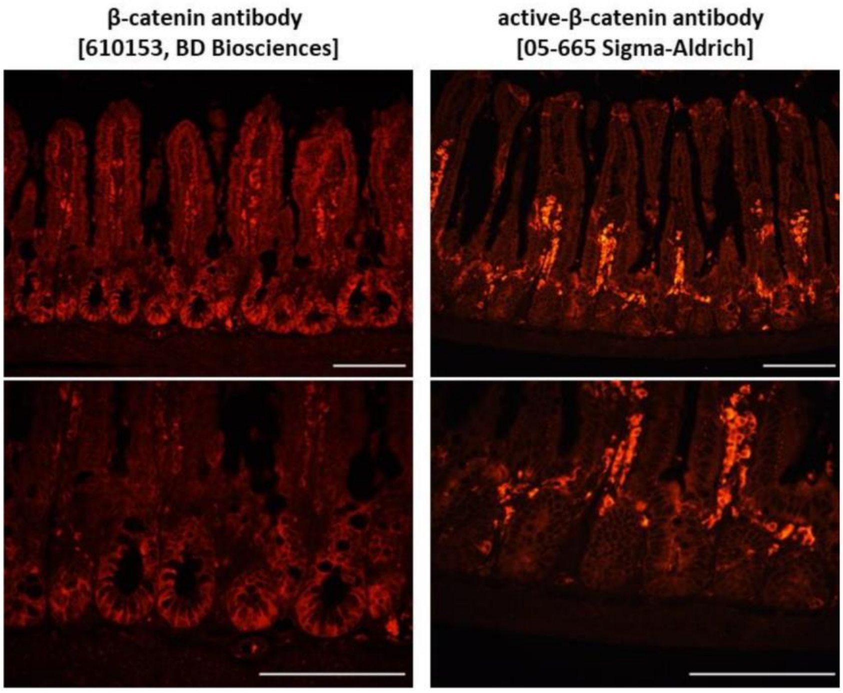

Reviewer 2 Advance Summary and Potential Significance to Field:
While a previous study (PMID 25892522) has already analyzed both conditional and whole body Fzd7 KO mice but described very different gut phenotypes from those found in the authors' KO mice. To improve the impact of the current study, the authors should further investigate the mechanisms underlying this discrepancy.

Response

Thank you for your valuable comment. This study (PMID 25892522) used three Fzd7 genetically modified mouse models, including two conditional knockout mice using AhCre (Cre recombinase driven by cytochrome P450 promoter) and Lgr5$^{EGFP-IRES-CreERT2}$ respectively and one conventional knockout mouse. They majorly studied the phenotypes of intestinal stem cells in mice at 6-12 weeks old but didn't show other phenotypes in intestines of mice. In addition, this article used these mouse models on different experiments respectively, we could not completely understand all phenotypes of their three mouse models, and therefore, could not compare the phenotypes of conventional mice between the study (PMID 25892522) and ours.
We modified the discussion in manuscript as followed description,

"Previous reports indicated that Fzd7 deletion in Lgr5-positive cells reduced the regenerative

© 2023. Published by The Company of Biologists under the terms of the Creative Commons Attribution License (https://creativecommons.org/licenses/by/4.0/).

capacity of the intestines (Flanagan et al., 2015). The Fzd7 genetically modified animal models in this article were two conditional knockout mice using AhCre (Cre recombinase driven by cytochrome P450 promoter; (Ireland et al., 2004)) and Lgr5$^{EGFP\text{-}IRES\text{-}CreERT2}$ respectively and one conventional knockout mouse at 6-12 weeks old. Flanagans and coleagues majorly investigated the effects of Fzd7 deletion on intestinal stem cells and indicated that Fzd7 plays an important role for robust Wnt-dependent processes in intestinal stem cells (Lgr5-positive cells). The models used in this article were analyzed around 12 weeks old and did not show any phenotypes related to enterocolitis. In human patients, the onset and progression of disease are induced by systemic problems, such as genetic defects or environmental stress. Enterocolitis is induced by an imbalance between proinflammatory and anti-inflammatory processes, disturbed epithelial homeostasis and a changed distribution of the microbiota. The Fzd7 conventional knockout model, generated in our lab, was analyzed from 3 weeks to 12 months old to confirm that Fzd7 deficiency causes severe enterocolitis and other phenotypes with time."

Reviewer 2 Comments for the Author:
The published work (PMID 25892522) has already analyzed both conditional and whole body Fzd7 KO mice, but these mice had distinct gut phenotypes from those found in the authors' KO mice. While the authors have addressed some aspects in their response letter, they still failed to discuss them in the revised manuscript. Given this earlier publication showing the conflicting data, the authors should perform additional studies to clarify the discrepancy, providing a clear explanation.

Response

Thank you for your valuable comment. This study (PMID 25892522) used three Fzd7 genetically modified mouse models, including two conditional knockout mice using AhCre (Cre recombinase driven by cytochrome P450 promoter) and Lgr5$^{EGFP\text{-}IRES\text{-}CreERT2}$ respectively and one conventional knockout mouse. They majorly studied the phenotypes of intestinal stem cells in mice at 6-12 weeks old but didn't show other phenotypes in intestines of mice. In addition, this article used these mouse models on different experiments respectively, we could not completely understand all phenotypes of their three mouse models, and therefore, could not compare the phenotypes of conventional mice between the study (PMID 25892522) and ours. We modified the discussion in manuscript as followed description,

"Previous reports indicated that Fzd7 deletion in Lgr5-positive cells reduced the regenerative capacity of the intestines (Flanagan et al., 2015). The Fzd7 genetically modified animal models in this article were two conditional knockout mice using AhCre (Cre recombinase driven by cytochrome P450 promoter; (Ireland et al., 2004)) and Lgr5$^{EGFP\text{-}IRES\text{-}CreERT2}$ respectively and one conventional knockout mouse at 6-12 weeks old. Flanagans and coleagues majorly investigated the effects of Fzd7 deletion on intestinal stem cells and indicated that Fzd7 plays an important role for robust Wnt-dependent processes in intestinal stem cells (Lgr5-positive cells). The models used in this article were analyzed around 12 weeks old and did not show any phenotypes related to enterocolitis. In human patients, the onset and progression of disease are induced by systemic problems, such as genetic defects or environmental stress. Enterocolitis is induced by an imbalance between proinflammatory and anti-inflammatory processes, disturbed epithelial homeostasis and a changed distribution of the microbiota. The Fzd7 conventional knockout model, generated in our lab, was analyzed from 3 weeks to 12 months old to confirm that Fzd7 deficiency causes severe enterocolitis and other phenotypes with time."

© 2023. Published by The Company of Biologists under the terms of the Creative Commons Attribution License (https://creativecommons.org/licenses/by/4.0/).

Many revised sentences in the text are unclear and require editing:
For example, line 163-164: "Consequently, beta-catenin was not activated and entered the nucleus of intestinal epithelial cells in Fzd7 KO mice (Supplemental Figure S4B)."

Response

Thank you for your comment. We changed the sentence as follow, "Our data indicated that β-catenin couldn't be activated and translocated into the nucleus of intestinal epithelial cells in Fzd7 KO mice (Fig. S4B)."

The authors claimed Fzd KO mice with chronic DSS injury developed rectal adenocarcinoma (main Fig 5G), but they stated that only 1 out of 8 mice had this phenotype. In order to claim rectal adenocarcinoma, they should increase sample size.

Response

Thank you for your comment. In this study, the chronic DSS injury was treated for three times and the intestines were harvested after the 90 days. In future, we will try a longer treatment to examine the tumorigenesis of Fzd7 KO mice with chronic injury.

We modified the sentences and the order of subfigures as followed description in manuscript,

"In addition, several phenotypes were also observed in Fzd7 KO mice given chronic DSS treatment, such as (a) diffuse mucosal and submucosal inflammatory infiltration and marked squamous metaplasia, and (b) marked mucosal and submucosal inflammatory infiltration with submucosal herniation and goblet cell depletion (Fig. 5G); moreover, Fzd7 deletion elevated the inflammatory score in the rectum (Fig. 5G). In our study, Fzd7 KO mouse with chronic DSS injury only for 90 days and had a potential to develop rectal adenocarcinoma (1/8= 12.5%) (Fig. 5H). Consequently, the combination of an intrinsic defect (Fzd7 deletion) and extrinsic factor (chronic DSS injury) seems to exhaust intestinal damage and contribute to tumorigenesis."

What is the sample size for liver tumor analysis of 12 month-old mice? What percentage of mice developed liver tumors? In addition, the authors still claimed that alterations in intestinal microbiota could induce tumorigenesis (line 264-266), but no functionally supporting data was provided.

Response

Thank you for your comment. Indeed, we didn't provide the functional data for that alterations in intestinal microbiota could induce tumorigenesis. In this manuscript, we want to emphasize the consequences of the epithelial defects which induce the intestinal diseases and contribute to liver pathogenesis through gut-liver axis. We also added the percentage of mice developed liver phenotypes in main text and showed the photos of phenotypes in Supplemental figure S9. We modified the sentences as followed description,

"As recent studies have shown that disturbances of intestinal microbiota and the metabolites were involved in the progression of chronic liver diseases (Llorente and Schnabl, 2015, Hartmann et al., 2019, Jones and Neish, 2021). These alterations of intestinal microbiotas could re-disturb the intestinal functions and enhance several features in liver, such as spotty necrosis (7/9=77.78%), portal inflammation (8/9=88.89%), piecemeal necrosis (6/9=66.67%), hepatocyte dysplasia (4/9=44.44%), focal coagulative necrosis (1/9=11.11%), fatty metamorphosis (2/9=22.22%) and further tumor growth (2/9=22.22%) in Fzd7 KO mice at 12 months old (Fig. 6E; Figs S9A-C); however, there was no significantly different histopathologic pattern in livers between Fzd7 KO and WT mice at 5, 7 and 9 months old (Figs S9D). Therefore, the imbalance of intestinal microbiota may not only destroy the intestinal integrity but induced the liver pathogenesis in Fzd7 KO mice."

© 2023. Published by The Company of Biologists under the terms of the Creative Commons Attribution License (https://creativecommons.org/licenses/by/4.0/).

Third decision letter

MS ID#: DEVELOP/2022/200932

MS TITLE: Frizzled-7 modulates Goblet and Paneth cell fate and maintains the homeostasis in intestines

AUTHORS: Nai-Xin Gu, Yu-Ru Guo, Sey-En Lin, Yen-Hsin Wang, I-Hsuan Lin, Yun Yen, and Yi-Fan Chen
ARTICLE TYPE: Research Article

I am happy to tell you that your manuscript has been accepted for publication in Development, pending our standard ethics checks.

© 2023. Published by The Company of Biologists under the terms of the Creative Commons Attribution License (https://creativecommons.org/licenses/by/4.0/).
